# Ca^2+^-associated triphasic pH changes in mitochondria during brown adipocyte activation

**DOI:** 10.1016/j.molmet.2017.05.013

**Published:** 2017-05-31

**Authors:** Yanyan Hou, Tetsuya Kitaguchi, Rókus Kriszt, Yu-Hua Tseng, Michael Raghunath, Madoka Suzuki

**Affiliations:** 1 WASEDA Bioscience Research Institute in Singapore (WABIOS), 11 Biopolis Way, #05-02 Helios, Singapore 138667, Singapore; 2 Comprehensive Research Organization, Waseda University, Tokyo, 162-0041, Japan; 3 Department of Biomedical Engineering, National University of Singapore, 117583, Singapore; 4 NUS Tissue Engineering Program, Life Sciences Institute, National University of Singapore, 117510, Singapore; 5 Graduate School for Integrative Sciences and Engineering (NGS), National University of Singapore, 117456, Singapore; 6 Section on Integrative Physiology and Metabolism, Joslin Diabetes Center, Harvard Medical School, Boston, MA 02215, USA; 7 Department of Biochemistry, Yong Loo Ling School of Medicine, National University of Singapore, 117597, Singapore; 8 PRESTO, Japan Science and Technology Agency (JST), 4-1-8 Honcho, Kawaguchi, Saitama, 332-0012, Japan

**Keywords:** Brown adipocytes, Ca^2+^, Confocal microscopy, Endoplasmic reticulum, Fluorescence imaging, Mitochondria-associated ER membrane, BAs, brown adipocytes, ISO, isoproterenol, ETC, electron transport chain, ER, endoplasmic reticulum, β-AR, β-adrenergic receptor, FFAs, free fatty acids, UCP1, uncoupling protein 1, Rot, rotenone, AMA, antimycin A, TMRM, tetramethylrhodamine methyl ester, IMS, intermembrane space, MAM, mitochondria-associated ER membrane, TG, thapsigargin, SERCA, sarco/endoplasmic reticulum Ca^2+^-ATPase, EGTA, ethylene glycol tetraacetic acid, MCU, mitochondrial calcium uniporter

## Abstract

**Objective:**

Brown adipocytes (BAs) are endowed with a high metabolic capacity for energy expenditure due to their high mitochondria content. While mitochondrial pH is dynamically regulated in response to stimulation and, in return, affects various metabolic processes, how mitochondrial pH is regulated during adrenergic stimulation-induced thermogenesis is unknown. We aimed to reveal the spatial and temporal dynamics of mitochondrial pH in stimulated BAs and the mechanisms behind the dynamic pH changes.

**Methods:**

A mitochondrial targeted pH-sensitive protein, mito-pHluorin, was constructed and transfected to BAs. Transfected BAs were stimulated by an adrenergic agonist, isoproterenol. The pH changes in mitochondria were characterized by dual-color imaging with indicators that monitor mitochondrial membrane potential and heat production. The mechanisms of pH changes were studied by examining the involvement of electron transport chain (ETC) activity and Ca^2+^ profiles in mitochondria and the intracellular Ca^2+^ store, the endoplasmic reticulum (ER).

**Results:**

A triphasic mitochondrial pH change in BAs upon adrenergic stimulation was revealed. In comparison to a thermosensitive dye, we reveal that phases 1 and 2 of the pH increase precede thermogenesis, while phase 3, characterized by a pH decrease, occurs during thermogenesis. The mechanism of pH increase is partially related to ETC. In addition, the pH increase occurs concurrently with an increase in mitochondrial Ca^2+^. This Ca^2+^ increase is contributed to by an influx from the ER, and it is further involved in mitochondrial pH regulation.

**Conclusions:**

We demonstrate that an increase in mitochondrial pH is implicated as an early event in adrenergically stimulated BAs. We further suggest that this pH increase may play a role in the potentiation of thermogenesis.

## Highlights

• A triphasic mitochondrial pH changes in adrenergically stimulated BAs was revealed.
• Phases 1 and 2 of the pH increase precede thermogenesis.
• The pH increase is partially related to electron transport chain activity.
• Ca^2+^ was transmitted from endoplasmic reticulum to mitochondria during phase 1.
• The transmitted Ca^2+^ regulates pH increase in mitochondria.

## 1 Introduction

Brown adipose tissue is capable of burning fat and could potentially counteract obesity-related metabolic diseases through specialized energy expenditure [1]. The discovery of brown adipose tissue in adult humans [2], [3], [4], [5] aroused great interest in understanding the regulatory pathways of brown adipocyte (BA) activation in the quest for strategies to promote energy expenditure [6]. The most well-known BA activation pathway is via the β-adrenergic receptor (β-AR) signaling pathway [1]. Upon binding to β-AR agonists, intracellular cAMP levels are elevated, which leads to the generation of free fatty acids (FFAs) in the cytoplasm [7]. FFAs subsequently bind to uncoupling protein 1 (UCP1), which activates its H^+^ conductance, resulting in H^+^ gradient dissipation accompanied by heat generation [7]. Apart from the known factors in the β-AR signaling pathway, several studies showed that Ca^2+^ is also involved in BA activation [8], [9], [10]. The increase in cytosolic Ca^2+^ levels enhances thermogenesis induced by β-AR signaling [9]. These studies imply regulatory networks of thermogenesis in BAs that are more complex than currently understood. Identifying new factors would provide additional targets for a synergistic control of energy expenditure.

Mitochondrial pH is an important physiological factor that influences various cellular activities [11]. In intact mitochondria, pH remains alkaline through the activity of the electron transport chain (ETC), with resting mitochondrial pH values ranging from 7.2 to 8.2 in different cell types [12]. Resting mitochondrial pH is disturbed in response to stimulation. For example, histamine stimulation, which induces both cytosolic and mitochondrial Ca^2+^ increases [13], leads to an increase in mitochondrial pH in HeLa cells [14]. In cortical neurons, glutamate stimulation results in an increase in mitochondrial pH [14], but this has also been described as transient and followed by a dramatic decrease in pH associated with an increase in cytosolic Ca^2+^ [15]. In isolated mitochondria, a pH increase can be induced by modulation of Ca^2+^ levels [16], [17]. Albeit not always in concordance, the literature points to a readily changed mitochondrial pH, especially by modulators of intracellular Ca^2+^ levels. Mitochondrial pH in return regulates various metabolic processes and even determines cell fate [18], [19].

How mitochondrial pH is regulated in stimulated BAs where the metabolic capacity is high [20] is unknown. To understand the dynamic regulation of mitochondrial pH in response to adrenergic stimulation, we used the pH sensitive protein mito-pHluorin, which was targeted to the mitochondrial matrix of BAs differentiated from murine adipocyte progenitors. The kinetics of mitochondrial pH changes were compared with the thermogenic process. In addition, we show that mitochondrial pH regulation is associated with changes in mitochondrial Ca^2+^ levels, which are further governed by the intracellular Ca^2+^ store that is the endoplasmic reticulum (ER).

## 2 Methods

### 2.1 Cell culture

Immortalized mouse brown preadipocytes (WT-1) [21] were cultured in high glucose Dulbecco's modified eagle medium (HG DMEM) supplemented with GlutaMAX (4 mM L-alanyl-l-glutamine dipeptide), 10% fetal bovine serum (FBS), 100 units/ml penicillin and 100 μg/ml streptomycin (P/S) at 37 °C in the presence of 5% CO_2_. All components were purchased from Thermo Fisher Scientific (MA, USA).

The differentiation of WT-1 has been previously described [22]. Briefly, WT-1 cells were seeded in 3.5 cm glass-based dishes (IWAKI, Tokyo, Japan) in basal medium [HG DMEM supplemented with GlutaMAX (4 mM L-alanyl-l-glutamine dipeptide), 2% FBS and P/S]. After reaching confluence, cells were treated with a differentiation cocktail (3.3 nM BMP-7, 20 nM insulin, and 1 nM T3 in basal medium) for three days. Cells were then treated with an induction cocktail (0.5 mM 3-isobutyl-1-methylxanthine (IBMX), 0.125 mM indomethacin, 5 μM dexamethasone, 20 nM insulin, and 1 nM T3 in basal medium) for two days. Finally, cells were maintained in maintenance medium (20 nM insulin and 1 nM T3 in basal medium) for another three days. Prior to use, cells were cultured in HG DMEM without FBS and hormonal supplements overnight. This procedure is to relieve the effects of the supplements on BAs in order to enable pronounced effect of ISO stimulation.

BMP-7 was purchased from R&D systems (MN, USA). Insulin, T3, IBMX, indomethacin and dexamethasone were purchased from Sigma–Aldrich (St. Louis, MO, USA).

### 2.2 Materials

Isoproterenol (ISO), rotenone (Rot), antimycin A (AMA), and Mdivi-1 were purchased from Sigma–Aldrich. NaN_3_ was purchased from BDH (Darmstadt, Germany). TMRM and thapsigargin were purchased from Thermo Fisher Scientific.

### 2.3 Plasmid construction and transfection

To generate mitochondrial matrix-localized green pH sensor, super ecliptic pHluorin [23] was fused to the C-terminus of the mitochondria targeting signal sequence from subunit VIII of cytochrome c oxidase: SVLTPLLLRGLTGSARRLPVPRAKIHSL. Mitochondrial targeting signal fused-pHluorin then replaced EGFP in the pEGFP-C3 vector, generating pmito-pHluorin-C3 (mito-pHluorin). To generate the endoplasmic reticulum (ER)-specific green pH sensor, super ecliptic pHluorin was inserted between the N-terminal calreticulin ER-targeting signal and the C-terminal KDEL ER-retention signal in a pcDNA3 vector, generating pcDNA3-ER-pHluorin (ER-pHluorin). Mitochondrial matrix-localized Ca^2+^ sensor CMV-mito-LAR-GECO1.2 (mito-R-GECO), and ER-localized Ca^2+^ sensor CMV-ER-LAR-GECO1 (ER-R-GECO) [24] were purchased from Addgene (MA, USA).

WT-1 was cultured and differentiated as described above. After induction, medium was changed to maintenance medium, and cells were transfected with plasmids using Lipofectamine 2000 (Thermo Fisher Scientific). After overnight incubation with transfection mixture, cells were exposed to maintenance medium for another two days and additional overnight incubation in supplement-free DMEM before imaging.

### 2.4 Confocal microscopy

Single-color imaging of mito-pHluorin or ER-pHluorin was performed with an Olympus FV1000 confocal microscope. A PlanApo N 60× NA 1.42 objective (Olympus) was used. Microscopic images were obtained with a 488 nm laser, a DM405/488/543 dichroic mirror, and a 555–655 nm emission band. Dual-color imaging was performed with an Olympus FV1200 confocal microscope. The same objective as FV1000 was used. Mito-pHluorin and ER-pHluorin images were acquired with a 473 nm laser, DM405/473/559/635 and SDM560 dichroic mirrors, and a BA490-540 emission filter. TMRM, ERthermAC, mito-R-GECO, and ER-R-GECO images were acquired with a 559 nm laser, a DM405/473/559/635 dichroic mirror and a BA575-675 emission filter. For imaging with fluorescent dyes, cells were incubated with 10 nM TMRM or 500 nM ERthermAC for 30 min at room temperature. DMEM supplemented with HEPES (Thermo Fisher Scientific) was used during dye incubation and subsequent imaging. Cells were maintained at 25 °C by a MIU-IBC-IF stage-top incubator (Olympus). For the treatments by ISO or other chemical drugs, 100 μl of solution was applied manually. To ensure that the pH changes during the process of ISO-induced BA activation was finely captured, cells were subjected to time lapse imaging with an interval of 20 s over 60 min or until cells were activated.

### 2.5 Titration of the pH dependence of pHluorin fluorescence intensity

The pH–intensity relationships of mito-pHluorin and ER-pHluorin were determined as previously described [25]. Briefly, transfected BAs were perfused with potassium-rich pH-clamping buffers (125 mM KCl, 20 mM NaCl, 10 mM Hepes, 10 mM MES (2-(N-morpholino)ethanesulfonic acid), 0.5 mM CaCl_2_, 0.5 mM MgCl_2_) containing 5 μM nigericin (an antibiotic drug that is able to equalize the pH across biological membranes, Sigma) with pH at 8.5, 8.0, 7.5, 7.0, 6.5, 6.0, 5.5, and 5.0, sequentially. The medium in the dish was removed using a peristaltic pump, followed by manually adding 2 mL of the fresh pH-clamping buffer. This sequence was repeated three times for each pH-clamping buffer. The fluorescence intensities at each pH point were acquired with a confocal microscope. Values of fluorescence intensity versus pH were plotted and fitted to a modified form of the Henderson–Hasselbalch equation:

$${\text{pH}}_{\text{x}}={\text{pK}}_{\text{a}}+{\text{log}}_{10}\left(\frac{\left({\text{If}}_{\text{x}}-{\text{If}}_{\text{min}}\right)}{\left({\text{If}}_{\text{max}}-{\text{If}}_{\text{x}}\right)}\right)\text{,}$$

where If_x_ is the fluorescence intensity measured at pH_x_. If_min_, If_max_, and pK_a_ are constants derived from the best fit curve.

To determine the physiological pH in mitochondria and ER, the fluorescence intensity was acquired before cells were perfused with pH-clamping buffers. Then the pH in each organelle was calculated using the aforementioned equation. The pH curves following ISO treatment were obtained using the same calibration curves respectively.

### 2.6 Data analysis and statistics

All fluorescence images were analyzed with ImageJ software (National Institute of Health, MD, USA). For time courses recording the relative fluorescence intensity and pH changes, a representative graph of one cell was shown. Each cell was recorded in an independent experiment using newly differentiated WT-1 cells. N refers to the number of cells/independent experiments quantified. Statistical results are presented as mean ± standard deviation (SD). Student's *t*-test was used for statistical analysis. n.s., not significant; *, p < 0.05; **, p < 0.01.

## 3 Results

### 3.1 Dynamic mitochondrial pH changes in BAs in response to β-adrenergic stimulation

**Figure 1:**
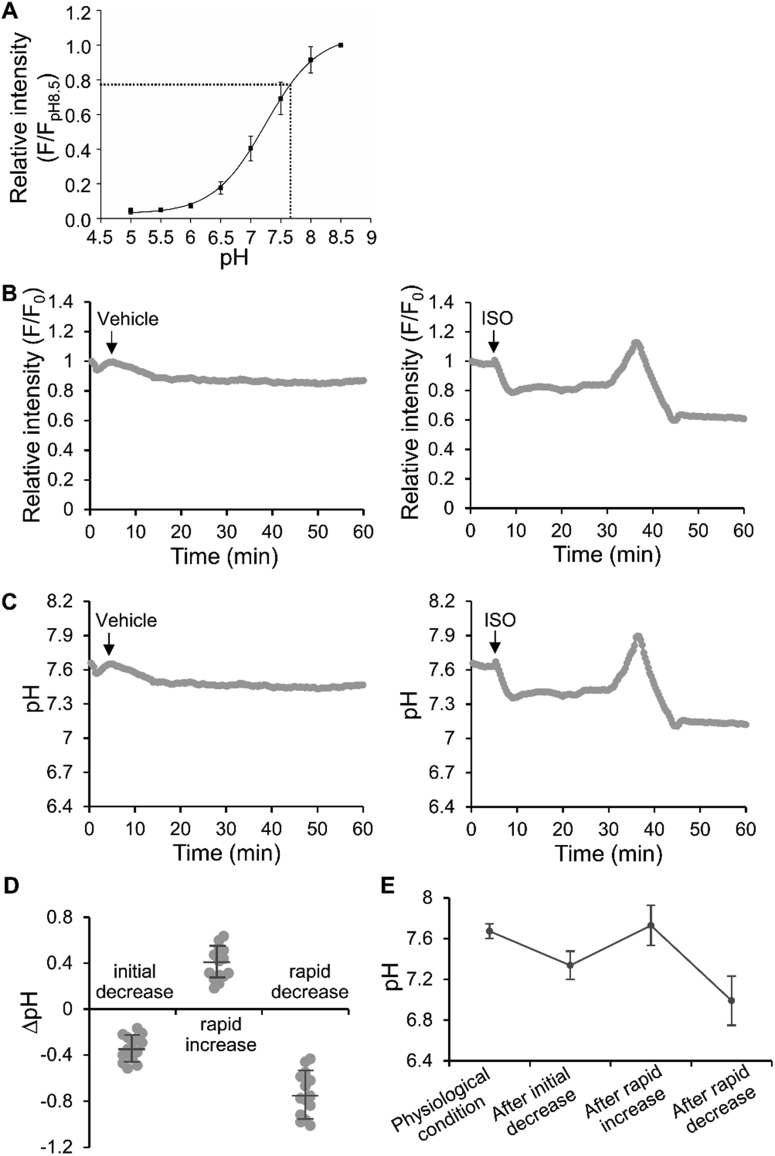
**Mitochondrial pH changes in BAs in response to ISO stimulation**. (**A**) Titration curve of mito-pHluorin in BAs determined under a confocal microscope. The curve is the best fit of the relative fluorescence intensity versus pH to Henderson–Hasselbalch equation. Dotted lines point to the relative fluorescence intensity and the corresponding mitochondrial pH under physiological conditions, 7.7 ± 0.2 (n = 13). (**B** and **C**) Representative time courses showing the changes in relative fluorescence intensity of mito-pHluorin (**B**) and the corresponding pH changes (**C**) in BAs following vehicle (left, n = 4) or ISO (right, n = 14) treatment over 60 min. See Movie S1. (**D** and **E**) Quantification of the relative (to the previous stage) (**D**) and the actual (**E**) mitochondrial pH changes in BAs following ISO treatment. Bars indicate means ± SD (n = 14).

Titration of mito-pHluorin revealed a p*K*_a_ of 7.2 in WT-1 differentiated BAs and that pH changes between 6.0 and 8.5 can be readily detected (Figure 1A). The calculated physiological pH in BA mitochondria was 7.7. To induce BA activation, we used isoproterenol (ISO), an agonist that non-selectively binds to β-adrenergic receptors and induces subsequent lipolysis and thermogenesis. After addition of 10 μM ISO, an initial decrease in fluorescence intensity of mito-pHluorin was immediately observed. The decreased intensity lasted for tens of minutes (17 ± 10 min, n = 14) before a rapid increase occurred, followed by a rapid decrease (Figure 1B, right and Movie S1, right). ISO treatment increases lipolysis [26] and oxidative phosphorylation [22]. These metabolic processes produce acidic products in the cytosol [27] and in the mitochondria [11], respectively. The initial pH decrease observed, therefore, was probably caused by enhanced metabolic activity before BAs were activated. In contrast, vehicle treatment did not elicit any significant intensity changes over 60 min in BAs (Figure 1B, left and Movie S1, left). Also, no significant intensity changes were observed in undifferentiated WT-1 cells over 60 min following ISO treatment (Figure S1 and Movie S2). The calculated pH changes in BAs following ISO treatment during the initial decrease, rapid increase, and rapid decrease were −0.3, 0.4, and −0.7, respectively (Figure 1C,D). It should be noted that the rapid decrease of −0.7 was probably an overestimate, as further explained in the Discussion section, Figure S2, and Movie S3. The resulting pH in BAs were 7.3, 7.7 and 7.0, respectively (Figure 1E).

Supplementary videos related to this article can be found at http://dx.doi.org/10.1016/j.molmet.2017.05.013.

The following are the supplementary video related to this article:

Movie S1 Mito-pHluorin fluorescence intensity changes in response to vehicle (left) or ISO (right) treatment in BAs, corresponding to time courses in Figure 1B, left and right, respectively. 20 μm × 29 μm (left) and 36 μm × 72 μm (right) (W × H). Movie S1

Movie S2 Mito-pHluorin fluorescence intensity changes in response to ISO treatment in undifferentiated WT-1 cells, corresponding to the time course in Figure S1A. 56 μm × 65 μm (W × H). Movie S2

Movie S3 Mito-EGFP (left) and tetramethylrhodamine methyl ester (TMRM, right) fluorescence intensity changes in response to ISO treatment in BAs, corresponding to time courses in Figure S2B. 52 μm × 56 μm (W × H). Movie S3

### 3.2 Delineating the three phases of mitochondrial pH changes

**Figure 2:**
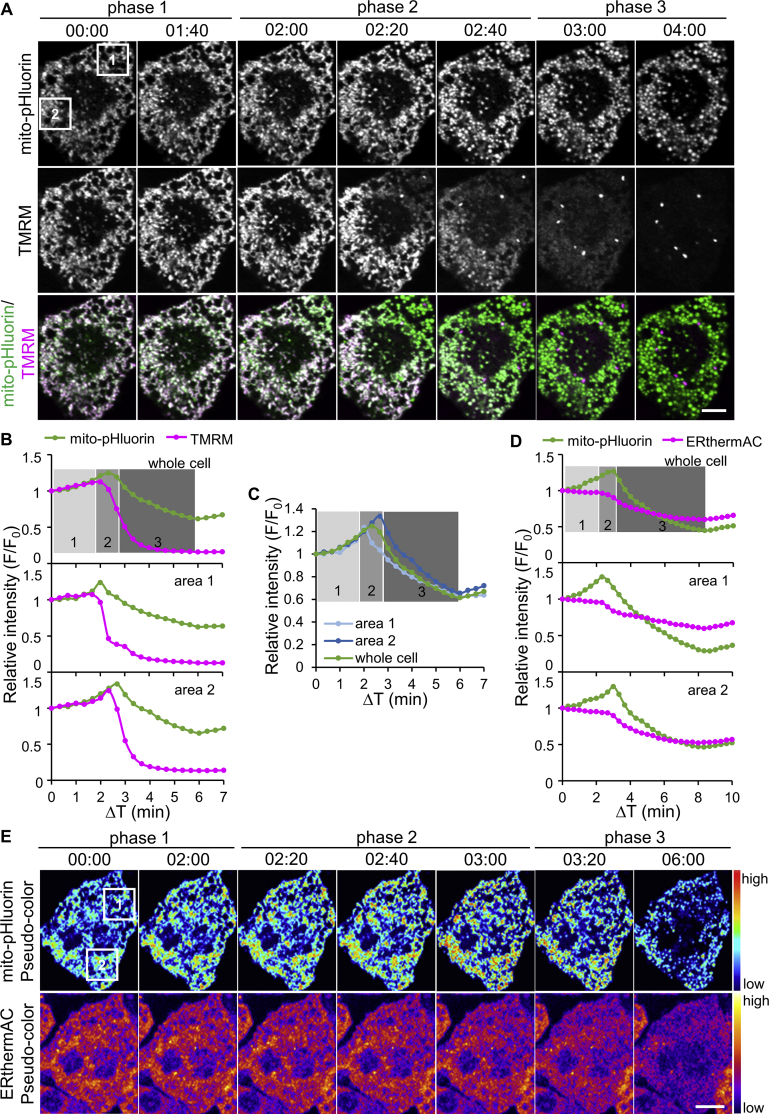
**Characterization of the triphasic pH changes in mitochondria**. (**A**) Images of a representative cell showing mito-pHluorin and tetramethylrhodamine methyl ester (TMRM, a fluorescent indicator of mitochondrial membrane potential ΔѰ_m_) intensity changes during the triphasic pH change (n = 9). The starting time point of pH increase was defined as t = 0 min. The wave of pH elevation propagated from top right to bottom left. Regions in squares were used for quantification in **B** and **C**. Scale bar, 10 μm. (**B**) Time courses showing the changes of the relative fluorescence intensities of mito-pHluorin and TMRM in the whole cell (upper), area 1 (middle) and area 2 (lower) of the cell shown in **A**. Different phases were marked with shaded areas on the graph (same for **C** and **D**). (**C**) Changes in the relative fluorescence intensity of mito-pHluorin in the whole cell, area 1, and area 2. (**D**) Representative time courses showing the changes of the relative fluorescence intensities of mito-pHluorin and ERthermAC during the triphasic pH change (n = 11). Note that the decrease in ERthermAC intensity occurred at the same time point as the decrease in mito-pHluorin intensity in phase 2, whereas the decrease in TMRM intensity started together with the increase in mito-pHluorin intensity in the same phase. (**E**) Pseudo-color images of the cell quantified in **D** showing mito-pHluorin and ERthermAC intensity changes. The wave of pH elevation propagated from top to bottom. Scale bar, 10 μm.

It is generally thought that the mitochondrial membrane potential (ΔѰ_m_) follows the H^+^ gradient. In other words, increased mitochondrial pH indicates more polarized mitochondria and vice versa. To evaluate whether the rapid pH increase indicated mitochondrial hyperpolarization and the pH decrease indicated depolarization, mito-pHluorin-transfected BAs were stained with tetramethylrhodamine methyl ester (TMRM), a red fluorescent dye that accumulates in polarized mitochondria. We found that the pH increase did indeed occur alongside a moderate increase in TMRM intensity (Figure 2A,B, phase 1), indicating that mitochondria became more polarized. Following this initial pH increase, a further pH elevation occurred in a wave pattern traversing individual cells from one side to the other (Figure 2A, phase 2). In contrast to the initial pH increase, mitochondrial depolarization, indicated by the loss of fluorescence intensity of TMRM, occurred during the wave of pH elevation (Figure 2A,B). In addition, the pH elevation wave was accompanied by mitochondrial fission and swelling. We confirmed that the intensity increase of mito-pHluorin in phase 2 was not caused by morphological changes of mitochondria by using a mitochondrial-localizing EGFP that is barely sensitive to pH in the range that occurs during phase 2 (see Discussion section, Figure S2, Movie S3). As the wave passed, the pH immediately started to decrease along the direction of the wave. The side where the wave began, therefore, reached peak pH earlier than the side where the wave ended (Figure 2C). The pH continued decreasing after the wave passed with further mitochondrial depolarization at a whole-cell level (Figure 2A,C, phase 3). According to these observations, we divided the ISO-induced pH changes in BAs into three phases: phase 1, pH increase with a moderately increased ΔѰ_m_; phase 2, wave of pH increase, mitochondrial depolarization and fission, followed by immediate pH decrease along the wave; phase 3, further pH decrease and mitochondrial depolarization.

Mitochondrial fission is required for BA activation [28]. To investigate the pH changes in fission-resistant cells, mito-pHluorin-transfected BAs were treated with a fission inhibitor, Mdivi-1 [29], [30]. Following Mdivi-1 treatment, mitochondria in BAs gradually became elongated (Figure S3A). Similar as in a previous report [28], Mdivi-1 treatment significantly reduced the percentage of BAs that were activated, indicated by mitochondrial depolarization (Figure S3B). Further, the mitochondrial pH in fission-resistant cells demonstrated an initial pH decrease following ISO treatment, while the triphasic pH changes did not occur (Figure S3C), suggesting that the triphasic pH changes in mitochondria only occurred in activated BAs.

BA activation, a process of UCP1-mediated H^+^ flow from the intermembrane space (IMS) back to the mitochondrial matrix, is accompanied by heat release. To confirm the timing of pH changes relative to BA activation, we stained mito-pHluorin transfected cells with ERthermAC [22], an ER-localized temperature-sensitive dye. Following ISO stimulation, a sudden decrease in ERthermAC intensity occurred, concurrent with the pH decrease during phase 2 that immediately followed the pH increase wave. The decrease in ERthermAC intensity, therefore, also followed a wave pattern, in the same direction as the pH increase wave propagated but with a time lag of a few seconds (Figure 2D,E). After the wave, the decrease in ERthermAC intensity continued alongside the phase 3 pH decrease at a whole-cell level (Figure 2D,E). This result indicated that the pH decrease in phases 2 and 3 corresponded to thermogenesis and hence the pH increase in phases 1 and 2 occurred before BA activation. What therefore causes the phase 1 and phase 2 pH increase?

### 3.3 The involvement of the ETC in inducing an increase in mitochondrial pH

**Figure 3:**
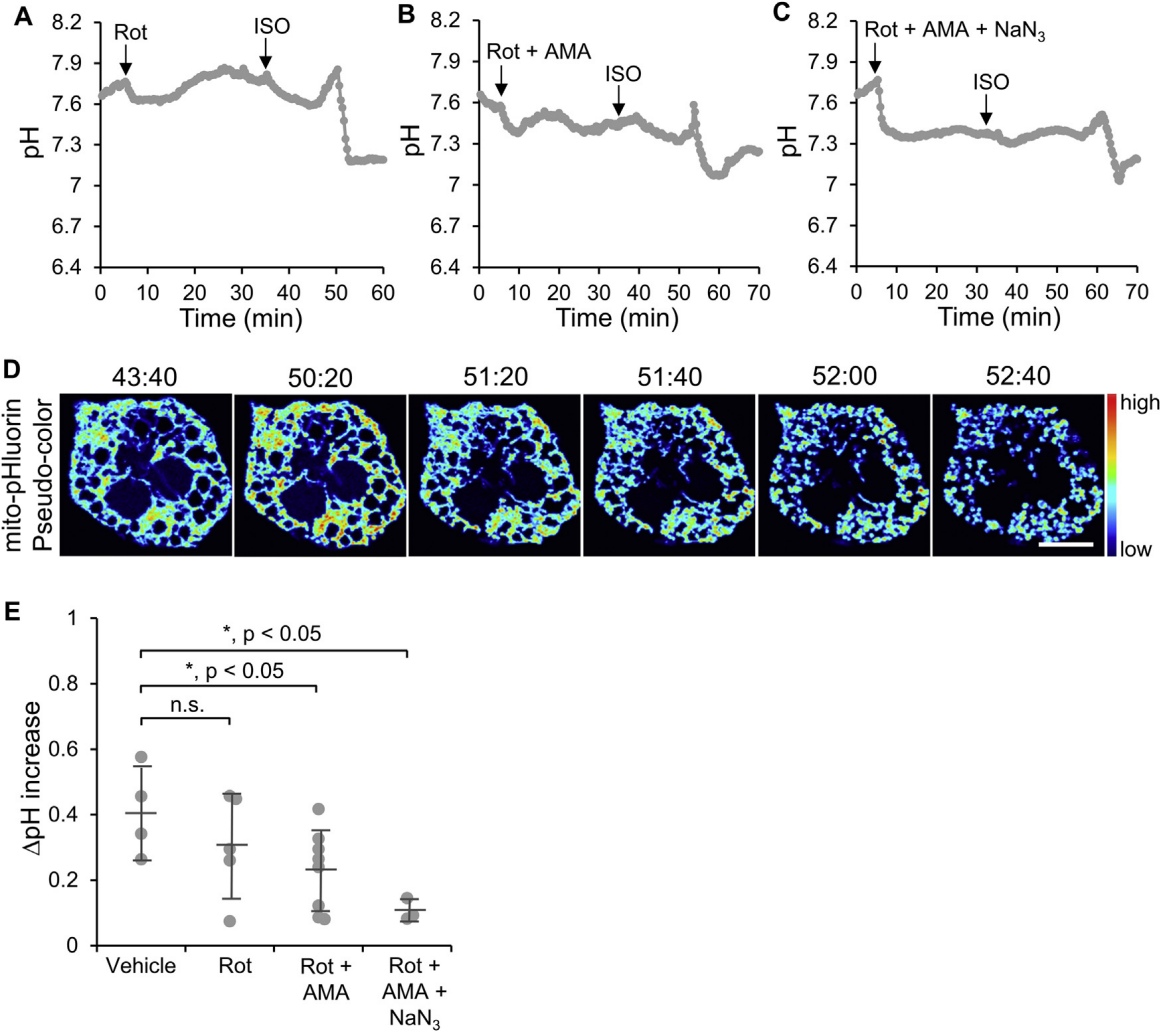
**The effect of electron transport chain (ETC) inhibitors on ISO-induced pH increase in BAs**. (**A**–**C**) Representative time courses showing the mitochondrial pH changes in response to 5 μM rotenone (Rot, **A**), 5 μM Rot + 5 μM antimycin A (AMA, **B**), and 5 μM Rot + 5 μM AMA + 3 mM NaN_3_ (**C**) treatments and subsequent ISO stimulation. (**D**) Pseudo-color images of the cell quantified in A showing mito-pHluorin intensity changes upon ISO stimulation with Rot-pre-treatment. See Movie S4, left. Scale bar, 10 μm. (**E**) Quantification and comparison of the relative pH increase in ISO-stimulated BAs pre-treated with 5 μM Rot (n = 5), 5 μM Rot + 5 μM AMA (n = 8), and 5 μM Rot + 5 μM AMA + 3 mM NaN_3_ (n = 3). Bars indicate means ± SD.

Given that the ETC mediates H^+^ transportation from the mitochondrial matrix to the IMS, an elevated mitochondrial pH could be induced by enhanced ETC activity. To test this hypothesis, rotenone (Rot), antimycin A (AMA), and NaN_3_, inhibitors that block the ETC complex I, complex III, and complex IV activities, respectively, were examined alone or in combination. Treatments with ETC inhibitors led to a pH decrease immediately after administration (Figure 3A–C), indicating that H^+^ transfer was inhibited. In the presence of ETC inhibitors, ISO treatment still elicited a pH increase in BAs, although to a lesser degree (Figure 3A–C). In addition, the wave pattern of pH increase disappeared and instead the pH increase occurred at a whole-cell level. The pH increase was followed by a pH decrease and mitochondrial fission, also at a whole-cell level (Figure 3D and Movie S4, left). The increases in pH were 0.3, 0.2, and 0.1 for Rot, Rot + AMA, and Rot + AMA + NaN_3_ treatment, respectively (Figure 3E), indicating that the ETC was involved in mediating H^+^ transfer during the ISO-induced increase in mitochondrial pH. However, the pH increase was not fully blocked by ETC inhibitors, even when all three H^+^ transfer complexes were inhibited, suggesting that other mechanisms that are capable of mediating H^+^ transportation were also involved.

Supplementary videos related to this article can be found at http://dx.doi.org/10.1016/j.molmet.2017.05.013.

The following is the supplementary video related to this article:

Movie S4 Mito-pHluorin (left) and mito-R-GECO (right) fluorescence intensity changes in response to 5 μM rotenone (Rot) treatment and subsequent ISO stimulation in BAs, corresponding to time courses in Figure 3A and Figure S5G, respectively. 31 μm × 29 μm (W × H). Movie S4

### 3.4 Concurrent mitochondrial Ca^2+^ and pH increase in response to β-adrenergic stimulation

**Figure 4:**
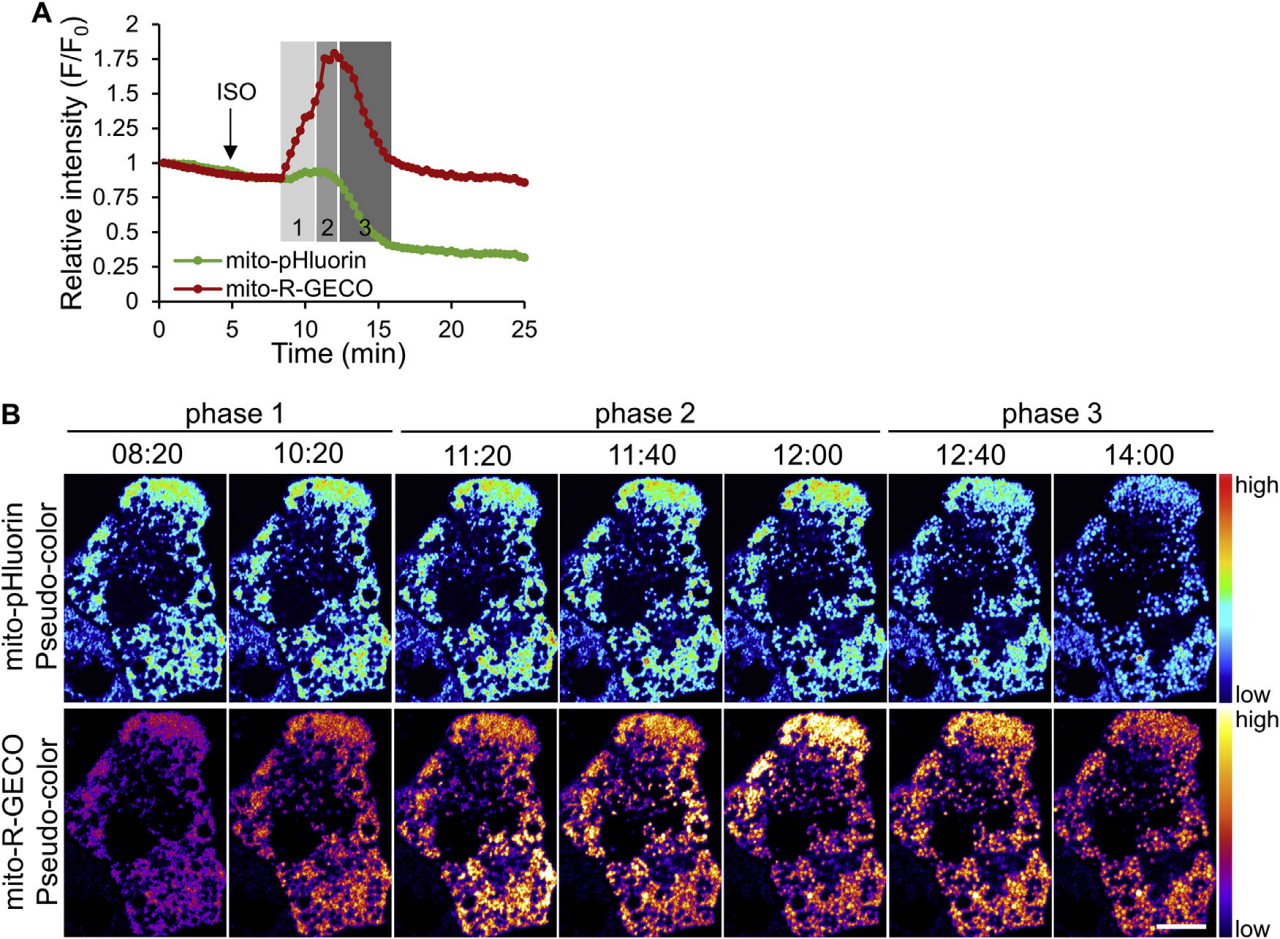
**ISO induces concurrent mitochondrial Ca**^**2+**^**and pH increases in BAs**. (**A**) Representative time courses showing the relative intensity changes of mito-pHluorin and mito-R-GECO in BAs following ISO stimulation (n = 5). Different phases are marked with shaded areas on the graph. (**B**) Pseudo-color images of the cell quantified in **A**. See Movie S5. Scale bar, 10 μm.

As suggested by the pH increase and mitochondrial depolarization during the wave in phase 2 (Figure 2A), it is plausible that H^+^ efflux from the mitochondrial matrix to the IMS was coupled to electrogenic reverse transportation of other cations. This would result in a net translocation of positive charges into the mitochondrial matrix and hence mitochondrial depolarization. Ca^2+^ has a role in regulating mitochondrial pH in many other cell types [13], [14], [15], [16], [17]. Therefore, we investigated if Ca^2+^ is also involved in the ISO-stimulated pH increase in BAs. To monitor mitochondrial pH and Ca^2+^ changes in the same cell, BAs were co-transfected with mito-pHluorin and a low-affinity Ca^2+^ indicator, mito-R-GECO [24]. ISO treatment induced mitochondrial Ca^2+^ increase in BAs, not only in phase 2 but also in phase 1 (Figure 4A). During phase 2, Ca^2+^ increase also followed a wave pattern, propagating from one end of the cell to another end, with the same timing as the pH increase (Figure 4B and Movie S5). The concurrent increase in mitochondrial pH and Ca^2+^ indicated a reverse transportation of H^+^ and Ca^2+^ during ISO stimulation.

Supplementary videos related to this article can be found at http://dx.doi.org/10.1016/j.molmet.2017.05.013.

The following is the supplementary video related to this article:

Movie S5 Mito-pHluorin (left) and mito-R-GECO (right) fluorescence intensity changes in response to ISO treatment in BAs, corresponding to time courses in Figure 4A. 33 μm × 47 μm (W × H). Movie S5

### 3.5 Involvement of Ca^2+^ transmission from ER to mitochondria in mitochondrial pH regulation

**Figure 5:**
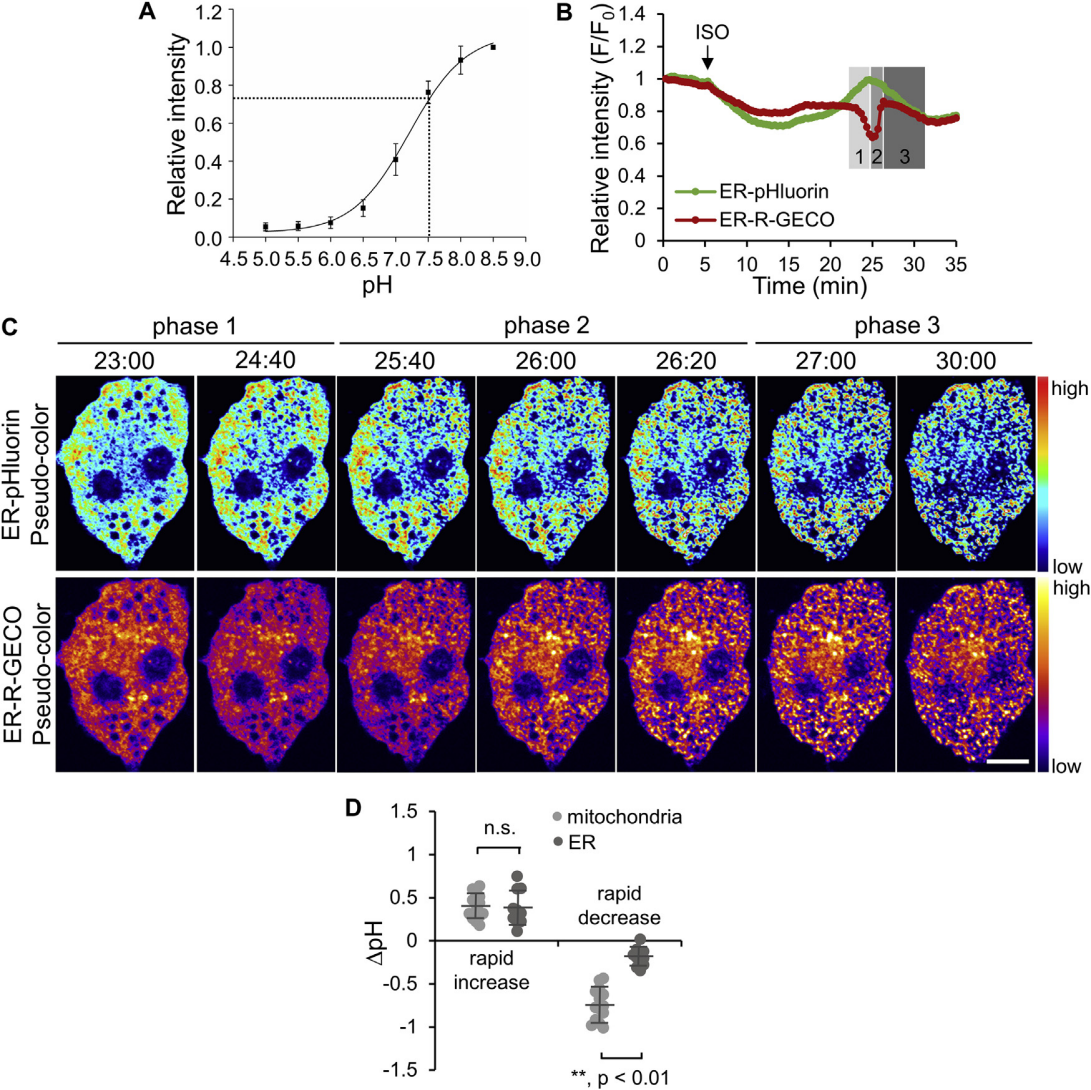
**pH and Ca**^**2+**^**changes in the ER in response to ISO stimulation**. (**A**) Titration curve of ER-pHluorin in BAs determined under a confocal microscope. Dotted lines point to the relative fluorescence intensity and the corresponding ER pH under physiological conditions, 7.6 ± 0.3 (n = 19). (**B**) Representative time courses showing the relative intensity changes of ER-pHluorin and ER-R-GECO in BAs following ISO stimulation. Different phases are marked with shaded areas on the graph. (**C**) Pseudo-color images of the cell quantified in B. See Movie S7. Scale bar, 10 μm. (**D**) Quantification and comparison of the pH changes between mitochondria (n = 14) and the ER (n = 10) in ISO-stimulated BAs. Bars indicate means ± SD. Data from mitochondria are reproduced from Figure 1D.

We further investigated the sources of the increased mitochondrial Ca^2+^ and their roles in regulating mitochondrial pH during ISO stimulation. In BAs, mitochondria are tethered to ER, an intracellular Ca^2+^ store [31], through the mitochondria-associated ER membrane (MAM) [32]. To understand the involvement of ER as the Ca^2+^ source for mitochondria, we examined the Ca^2+^ as well as pH changes in ER following ISO stimulation. BAs were co-transfected with an ER-localized low-affinity Ca^2+^ indicator, ER-R-GECO [24], and an ER-localized pHluorin, ER-pHluorin (p*K*_a_ 7.2, Figure 5A). The changes in Ca^2+^ and pH in ER were also divided into three phases by comparing the pH changes to ΔѰ_m_ using dual-color imaging of ER-pHluorin and TMRM (Figure S4 and Movie S6). Following ISO stimulation, the ER showed a rapid decrease in Ca^2+^ during phase 1, followed by a rapid increase in phase 2 (Figure 5B,C and Movie S7, right). This is in contrast to the monotonic increase in both phases of mitochondrial Ca^2+^. The opposing changes in Ca^2+^ between the ER and mitochondria during phase 1 probably indicate Ca^2+^ transmission from the ER to mitochondria. The physiological pH within ER was estimated to be 7.6, which is close to the physiological pH in mitochondria (Figure 1A). Following ISO stimulation, ER pH showed an increase in phase 1 to a similar extent as that of the pH increase in mitochondria (Figure 5D), although without a remarkable increase in phase 2 (Figure 5B,C and Movie S7, left). Similar to mitochondrial fission, the ER also showed a pattern of fragmentation during phase 2 (Figure 5C). The pH decrease in phase 3 is considerably smaller than that in mitochondria (Figure 5D), highlighting the role of UCP1 in mediating H^+^ transportation in mitochondria.

Supplementary videos related to this article can be found at http://dx.doi.org/10.1016/j.molmet.2017.05.013.

The following are the supplementary videos related to this article:

Movie S6 ER-pHluorin (left) and TMRM (right) fluorescence intensity changes in response to ISO treatment in BAs, corresponding to time courses in Figure S4A. 43 μm × 53 μm (W × H). Movie S6

Movie S7 ER-pHluorin (left) and ER-R-GECO (right) fluorescence intensity changes in response to ISO treatment in BAs, corresponding to time courses in Figure 5B. 33 μm × 46 μm (W × H). Movie S7

**Figure 6:**
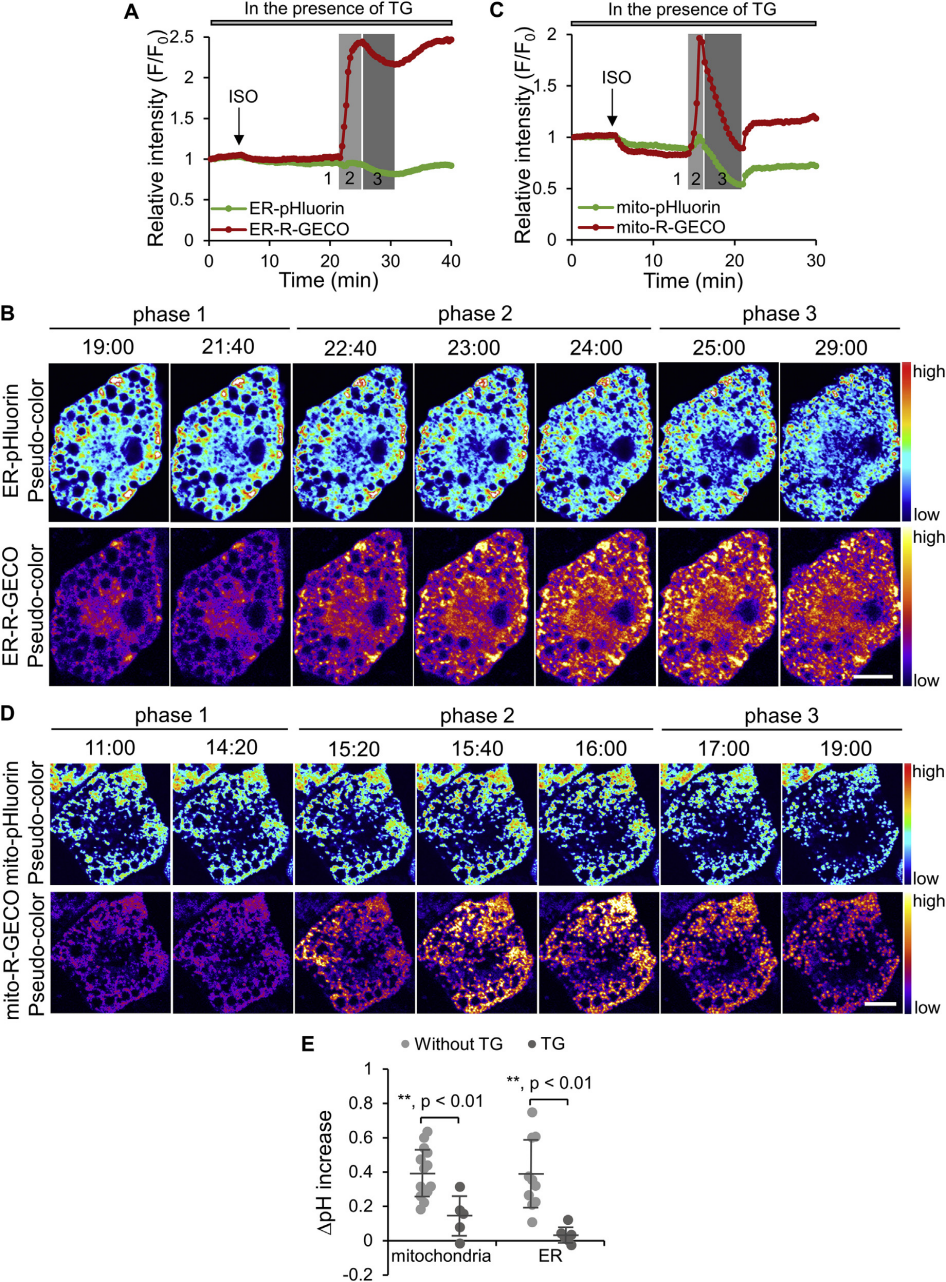
**The effect of thapsigargin (TG) on ISO-induced Ca**^**2+**^**and pH changes in the ER and mitochondria**. (**A**, **C**) Representative time courses showing the relative intensity changes of ER-pHluorin and ER-R-GECO (**A**) and mito-pHluorin and mito-R-GECO (**C**) following ISO stimulation in BAs pre-treated with 2 μM TG for 30 min. Different phases are marked with shaded areas on the graph. Phase 1 could not be identified due to the lack of a starting point. (**B**, **D**) Pseudo-color images of cells quantified in **A** and **C**, respectively. See Movies S8 (for **B**) and S9 (for **D**). Scale bars, 10 μm. (**E**) Quantification and comparison of the pH changes in ISO-stimulated BAs without and with TG treatment. Data are means ± SD (mitochondria: n = 14 without TG, n = 5 with TG; ER: n = 10 without TG, n = 6 with TG).

Ca^2+^ transmission from the ER to mitochondria in phase 1 was further supported by depleting Ca^2+^ in the ER using thapsigargin (TG), a sarco/endoplasmic reticulum Ca^2+^-ATPase (SERCA) inhibitor that blocks Ca^2+^ uptake into the ER from cytosol (Figure S5A). In mitochondria, TG incubation did not significantly affect Ca^2+^ levels over 30 min (Figure S5B), which was in contrast to previous reports that TG treatment induces mitochondrial Ca^2+^ increase through mitochondrial calcium uniporter (MCU) [33], [34], [35]. The insignificant Ca^2+^ increase in BAs was probably due to a low MCU activity in metabolically active cells [36] (cf. Discussion). Following a 30 min pre-incubation with TG, BAs were subjected to ISO stimulation in the presence of TG. As expected, ISO was not able to elicit a decrease in Ca^2+^ in the ER in phase 1 (Figure 6A,B and Movie S8, right). However, phase 2 still showed a marked Ca^2+^ increase. Similar to what was observed in the ER, ISO also failed to elicit the phase 1 Ca^2+^ increase in mitochondria and directly induced the phase 2 Ca^2+^ increase wave in the presence of TG (Figure 6C,D and Movie S9, right). TG treatment also abolished the phase 1 pH increase, in both mitochondria and ER (Figure 6A,C and Movies S8, left and S9, left). In the presence of TG, the ISO-induced pH increase in mitochondria and the ER were 0.1 and 0.0, respectively (Figure 6E), which were significantly lower than those in non-treated cells. Together, these results suggest that Ca^2+^ is transmitted from the ER into mitochondria in ISO-stimulated BAs and that the transmitted Ca^2+^ is involved in pH regulation within both organelles.

Supplementary videos related to this article can be found at http://dx.doi.org/10.1016/j.molmet.2017.05.013.

The following are the supplementary videos related to this article:

Movie S8 ER-pHluorin (left) and ER-R-GECO (right) fluorescence intensity changes in response to ISO treatment in BAs pre-treated with 2 μM thapsigargin (TG), corresponding to time courses in Figure 6A. 31 μm × 41 μm (W × H). Movie S8

Movie S9 Mito-pHluorin (left) and mito-R-GECO (right) fluorescence intensity changes in response to ISO treatment in BAs pre-treated with 2 μM TG, corresponding to time courses in Figure 6C. 38 μm × 38 μm (W × H). Movie S9

## 4 Discussion

In this study, we revealed a triphasic mitochondrial pH change during ISO-induced BA activation. While the phase 3 pH decrease corresponded to thermogenesis, the phase 1 and phase 2 pH increases occurred before thermogenesis, with concurrent increases in mitochondrial Ca^2+^. Cytosolic Ca^2+^ increase has been reported in BAs upon adrenergic stimulation in several studies [8], [10], [37], [38]. It was reported that cytosolic Ca^2+^ increase potentiates thermogenesis induced by β-AR signaling [9]. We reported here that an increase in Ca^2+^ also occurred in mitochondria, immediately before the initiation of the thermogenic process, and that this increase is implicated in the regulation of mitochondrial pH.

We confirmed that the increase in intensity of mito-pHluorin in phase 2 was not caused by morphological changes in mitochondria by using mitochondrial-localizing EGFP. Titration showed that mito-EGFP is only slightly sensitive to pH, with a 2.8% increase in intensity between pH 7.3 to 7.8, the pH range of phase 2 (Figure S2A). In a cell expressing mito-EGFP (Figure S2B), TMRM exhibited a similar response as that observed in mito-pHluorin transfected BAs (Figure 2A). Therefore, we considered that the moderate intensity increase in TMRM corresponded to phase 1 and was followed by phase 2, in which a drastic decrease in TMRM intensity took place during the fission wave (Figure S2B). During this mitochondrial fission and depolarization wave in phase 2, no significant increase in mito-EGFP fluorescence intensity was observed in the cell (Figure S2B,C and Movie S3), implying that fluorescence intensity was not affected by mitochondrial fission-induced morphological changes. The increase of mito-pHluorin intensity during the fission wave therefore probably reflected a pH increase. After mitochondrial fission, i.e. in phase 3, mito-EGFP showed a noticeable decrease in intensity of about 20% (Figure S2B). During this phase H^+^ leak and thermogenesis occurs; the decrease in mito-EGFP intensity could therefore be attributed to its modest pH sensitivity in the range of pH 7.0 to 8.0 with a 6.5% increase in intensity and its thermosensitivity of about – 1%/°C [39]. Considering these factors, the extent of the rapid decrease in pH during phase 3 estimated from the intensity of mito-pHluorin (Figure 1D), which has a temperature sensitivity of – 1.4%/°C (Figure S2D), therefore, may be overestimated. And the overestimation was insignificantly contributed by the photobleaching of mito-pHluorin (Figure S2E).

When the ER was depleted of Ca^2+^, mitochondria still showed an increase in Ca^2+^ during phase 2, suggesting that the extracellular environment, and not the ER, is the Ca^2+^ source during this phase. This hypothesis was tested using Ca^2+^-free medium supplemented with 5 mM ethylene glycol tetraacetic acid (EGTA). To avoid the detrimental effects of EGTA on BAs over long-term incubation, EGTA medium was added after ISO-induced phase 1 Ca^2+^ changes occurred. After addition of EGTA, the increase in Ca^2+^ in both mitochondria (Figure S5C,D and Movie S10, left) and the ER (Figure S5E,F and Movie S10, right) during phase 2 was markedly suppressed, suggesting that the Ca^2+^ uptake into both mitochondria and the ER during this phase was from the extracellular environment. Apart from the differences in Ca^2+^ sources, phase 2 pH and Ca^2+^ increases both exhibited wave patterns in mitochondria. As the wave of pH and Ca^2+^ disappeared when cells were treated with ETC inhibitors (Figure 3D and S5H and Movie S4), we can conclude that the wave generation in BAs is dependent on an established H^+^ gradient. Other differences between phase 1 and phase 2 include changes in ΔѰ_m_ and mitochondrial morphology. Specifically, a decrease in ΔѰ_m_, mitochondrial fission, and swelling only began in phase 2, despite an increase in mitochondrial Ca^2+^ occurring in both phases. During the Ca^2+^ wave in phase 2, it is probable that the local Ca^2+^ concentration is high while the wave passes through. This transiently high Ca^2+^ concentration may result in a net translocation of positive charges into the mitochondrial matrix, leading to a decrease in ΔѰ_m_. In addition, the high mitochondrial Ca^2+^ concentration induces fragmentation [40] and may also lead to osmosis, explaining the mitochondrial swelling during this phase. Therefore, the differences in ΔѰ_m_ and mitochondrial morphology between phases 1 and 2 probably represent a difference in the amplitude of Ca^2+^ increase in mitochondria.

Supplementary videos related to this article can be found at http://dx.doi.org/10.1016/j.molmet.2017.05.013.

The following is the supplementary video related to this article:

Movie S10 Mito-R-GECO (left) and ER-R-GECO (right) fluorescence intensity changes in response to ISO treatment and subsequent EGTA perfusion, corresponding to time courses in Figure S5C and E, respectively. 40 μm × 47 μm (left) and 31 μm × 46 μm (right) (W × H). Movie S10

In BAs, mitochondria are physically tethered to the ER through MAM [32]. MAM has been reported in other cell types where it facilitates Ca^2+^ transmission from the ER to mitochondria [41], [42] and therefore influences the biological processes in mitochondria and even determines cell fate [43]. Our result suggests a similar role of MAM in facilitating Ca^2+^ transmission from the ER to mitochondria in BAs, as evidenced by mitochondria and the ER exhibiting opposing Ca^2+^ level changes during phase 1. On the other hand, MAM may promote reverse Ca^2+^ transmission from mitochondria to the ER, hence Ca^2+^ refilling of the ER when its Ca^2+^ levels are low. This concept could be supported by the result that the ER still showed a marked Ca^2+^ increase in phase 2 when SERCA, the sole Ca^2+^ pump on the ER membrane, was blocked using TG. MAM may therefore function as a bidirectional link between mitochondria and the ER in stimulated BAs.

Besides Ca^2+^ transmission, MAM also showed an impact on pH regulation in these two organelles. In unstimulated BAs, mitochondria and ER exhibited a similar pH under physiological conditions, a phenomenon that is different from many other cell types, which show a pH difference of about 0.5–1.0 pH unit between mitochondria and the ER [11], [44]. In stimulated BAs, both mitochondria and the ER showed a pH increase in phase 1. As the pH increased, both compartments showed accompanied changes in Ca^2+^ levels. The pH could therefore be directly and separately regulated by Ca^2+^ level changes in each compartment, i.e. the ER pH increase is regulated by a Ca^2+^ decrease in the ER and the mitochondrial pH increase is regulated by a Ca^2+^ increase in mitochondria. However, it was reported that the pH of the ER is not affected by Ca^2+^ release from the ER [45], suggesting that its pH was probably indirectly influenced by the mitochondrial pH. The tight association between the ER and mitochondria may therefore regulate the physiological parameters of these organelles in both resting and stimulated conditions in BAs.

**Figure 7:**
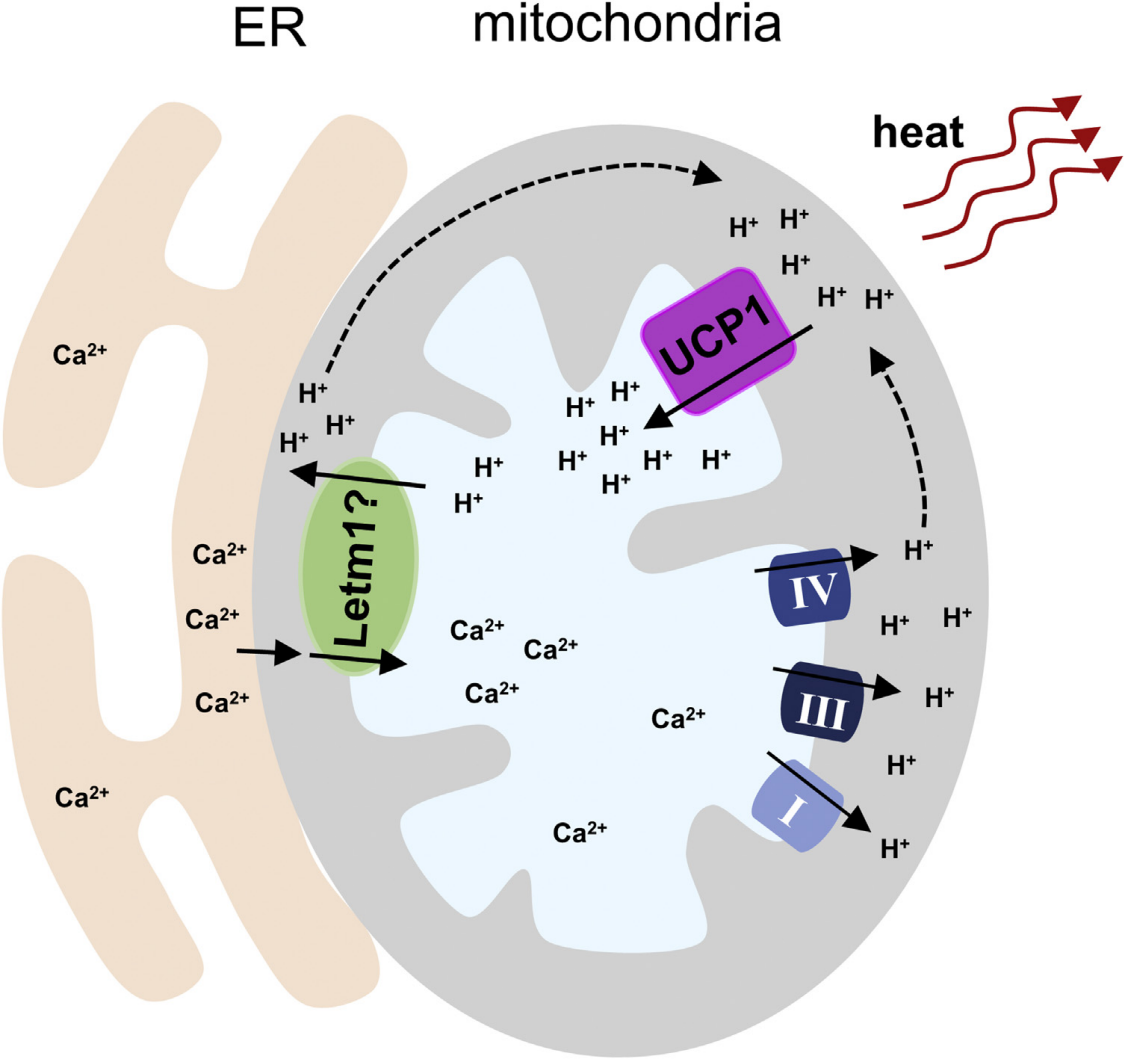
Schematic of Ca^2+^-regulated mitochondrial pH increase during ISO-induced BA thermogenesis.

Earlier studies by Denton and McCormack showed that Ca^2+^ activates four mitochondrial dehydrogenases, resulting in increased NADH production and enhanced ETC activity [46]. With the latter, more H^+^ are expected to be transported out of the mitochondrial matrix, leading to an increased mitochondrial pH. It is therefore probable that the increased mitochondrial pH resulted in part from enhanced ETC activity stimulated by increased mitochondrial Ca^2+^ levels. Indeed, treatment with ETC inhibitors suppressed the amplitude of the pH increase in mitochondria. However, inhibitors did not fully block the pH rise in ISO-stimulated BAs, suggesting that other H^+^ transportation mechanisms are also involved in this process. Recently, a mitochondrial Ca^2+^/H^+^ antiporter Letm1, which mediates Ca^2+^ uptake into the mitochondrial matrix and H^+^ extrusion to the IMS [47], [48], [49], was identified using genome-wide RNA interference screening [50]. It was estimated that Letm1 mediates a 1:1 electrogenic Ca^2+^/H^+^ exchange [50]. Mitochondrial Ca^2+^, therefore, may regulate mitochondrial pH through a combination of two different mechanisms (Figure 7). We speculated that in BAs, Letm1 rather than MCU is the primary mechanism for mitochondrial uptake of Ca^2+^. Although MCU has been widely accepted as the main source of mitochondrial Ca^2+^ uptake in many cell types [34], [51], [52], it has been reported that the activity of MCU varies significantly between tissues [36]. Specifically, the activity of MCU in metabolically active tissues is dramatically low or barely detectable [36], which is believed to prevent mitochondrial Ca^2+^ overload as the cytosolic Ca^2+^ signaling in those tissues is intensive. BAs are metabolically active cells and are probably also endowed with a low MCU activity. However, the function of Letm1 is still under debate. Other studies have suggested that Letm1 is also a transporter of other cations such as K^+^ [49], [53] and that the transportation is electroneutral [49]. The presence and function of Letm1 in BAs should therefore be studied specifically in this cell type in order to clarify its involvement in the ISO-induced increase in mitochondrial pH.

In summary, this study presents a Ca^2+^-regulated pH increase in mitochondria immediately before thermogenesis in stimulated BAs. As the amount of heat produced during thermogenesis is directly linked to the electrochemical energy stored in the H^+^ gradient across the inner mitochondrial membrane, the increase in mitochondrial pH probably functions as a mechanism to potentiate thermogenesis in BAs. In addition, we also presented a wave pattern of pH and Ca^2+^ increase in mitochondria. Ca^2+^ waves in cytosol have been observed frequently in oocytes [54], [55] and in neurons [56] with the function of providing development signals and activating Ca^2+^-dependent membrane conductance [56], respectively. In BAs, as the pH and Ca^2+^ wave was immediately followed by thermogenesis, the wave pattern is probably associated with the thermogenic function of BAs.
