# Supplementary figures and images for: Ca2+-associated triphasic pH changes in mitochondria during brown adipocyte activation
Source: Mol Metab. 2017 May 31;6(8):797–808. doi: 10.1016/j.molmet.2017.05.013 (PMC5518710; doi:10.1016/j.molmet.2017.05.013)

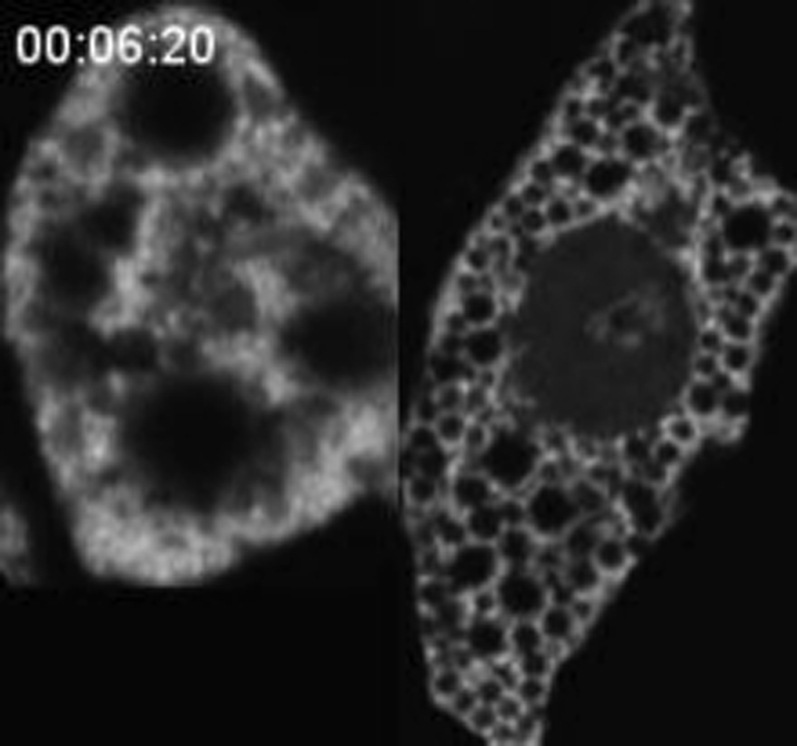

Supplement: Movie S1 — Mito-pHluorin fluorescence intensity changes in response to vehicle (left) or ISO (right) treatment in BAs, corresponding to time courses in Figure 1B, left and right, respectively. 20 μm × 29 μm (left) and 36 μm × 72 μm (right) (W × H). [file mmc2.jpg]

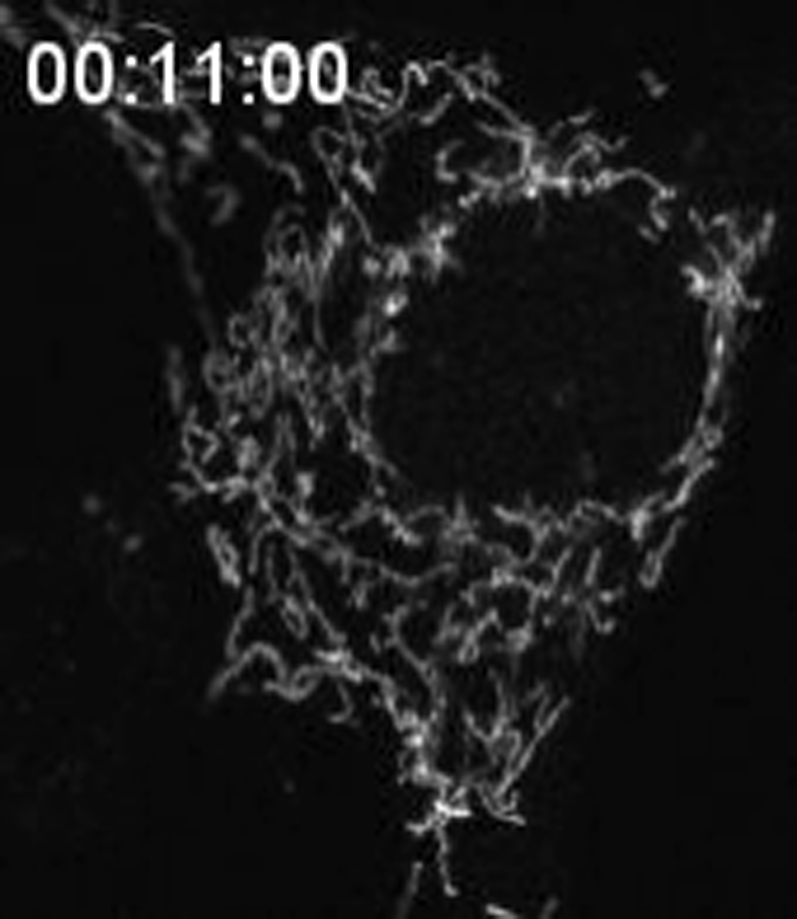

Supplement: Movie S2 — Mito-pHluorin fluorescence intensity changes in response to ISO treatment in undifferentiated WT-1 cells, corresponding to the time course in Figure S1A. 56 μm × 65 μm (W × H). [file mmc3.jpg]

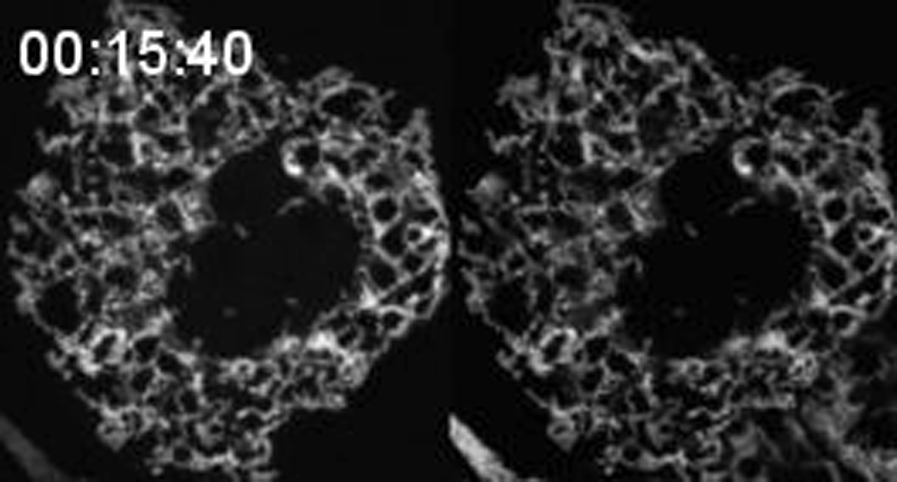

Supplement: Movie S3 — Mito-EGFP (left) and tetramethylrhodamine methyl ester (TMRM, right) fluorescence intensity changes in response to ISO treatment in BAs, corresponding to time courses in Figure S2B. 52 μm × 56 μm (W × H). [file mmc4.jpg]

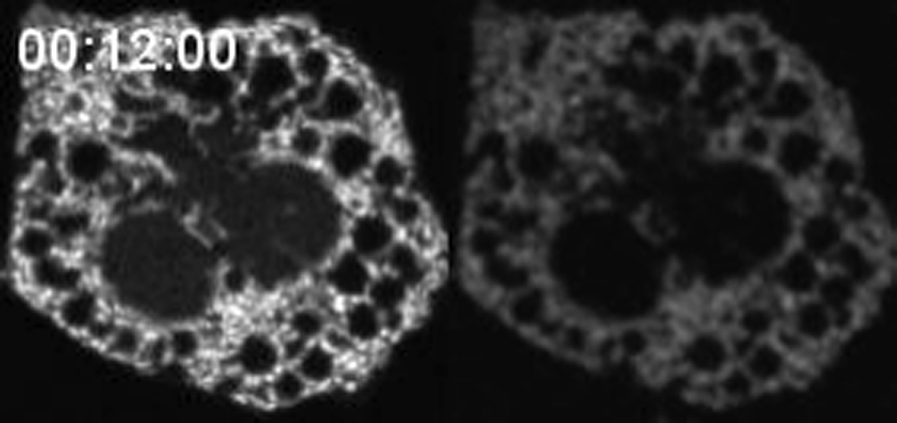

Supplement: Movie S4 — Mito-pHluorin (left) and mito-R-GECO (right) fluorescence intensity changes in response to 5 μM rotenone (Rot) treatment and subsequent ISO stimulation in BAs, corresponding to time courses in Figure 3A and Figure S5G, respectively. 31 μm × 29 μm (W × H). [file mmc5.jpg]

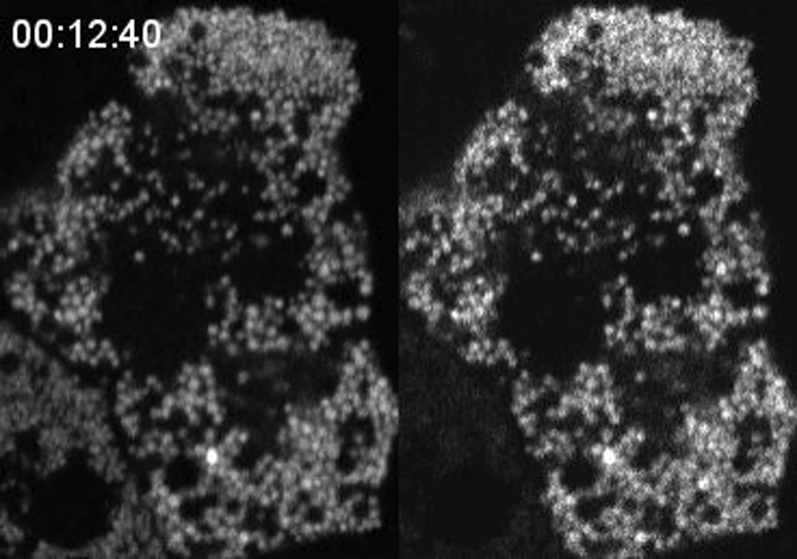

Supplement: Movie S5 — Mito-pHluorin (left) and mito-R-GECO (right) fluorescence intensity changes in response to ISO treatment in BAs, corresponding to time courses in Figure 4A. 33 μm × 47 μm (W × H). [file mmc6.jpg]

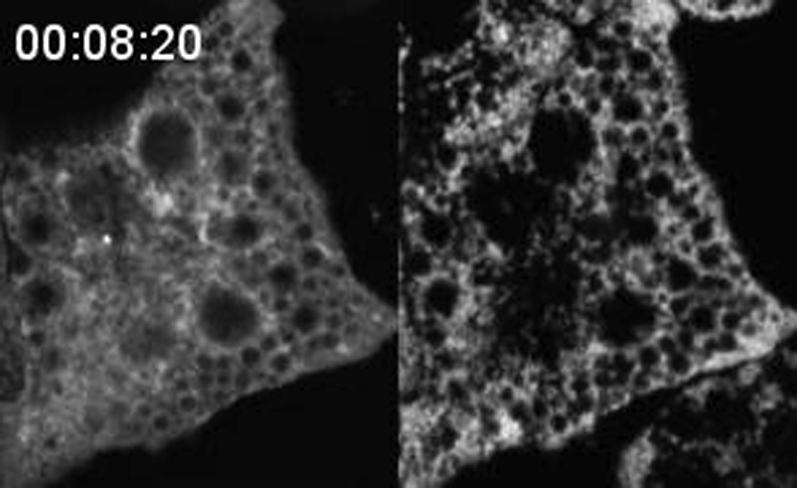

Supplement: Movie S6 — ER-pHluorin (left) and TMRM (right) fluorescence intensity changes in response to ISO treatment in BAs, corresponding to time courses in Figure S4A. 43 μm × 53 μm (W × H). [file mmc7.jpg]

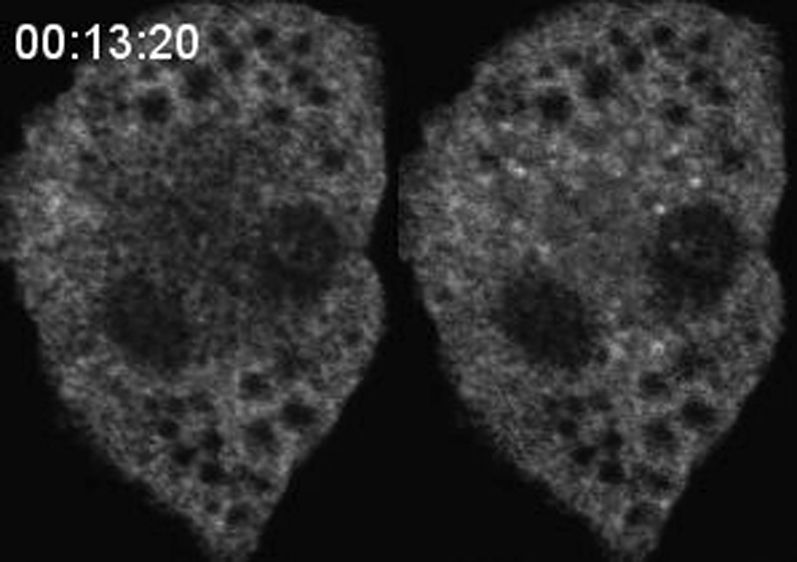

Supplement: Movie S7 — ER-pHluorin (left) and ER-R-GECO (right) fluorescence intensity changes in response to ISO treatment in BAs, corresponding to time courses in Figure 5B. 33 μm × 46 μm (W × H). [file mmc8.jpg]

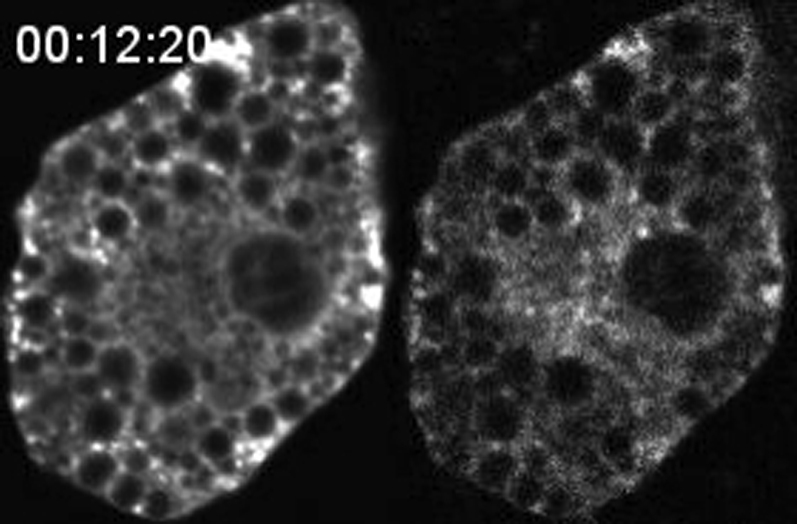

Supplement: Movie S8 — ER-pHluorin (left) and ER-R-GECO (right) fluorescence intensity changes in response to ISO treatment in BAs pre-treated with 2 μM thapsigargin (TG), corresponding to time courses in Figure 6A. 31 μm × 41 μm (W × H). [file mmc9.jpg]

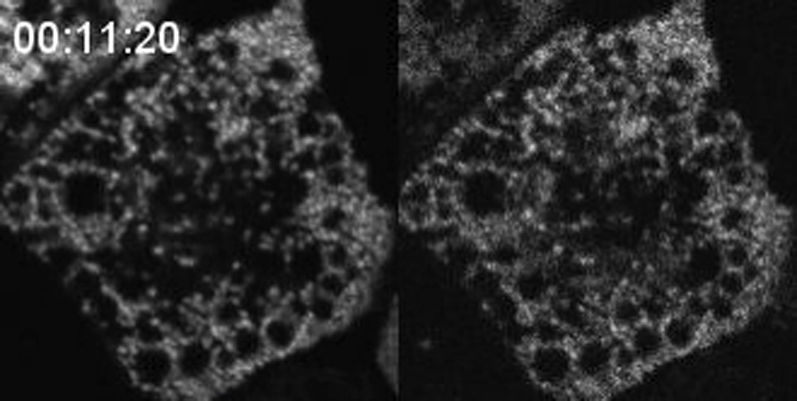

Supplement: Movie S9 — Mito-pHluorin (left) and mito-R-GECO (right) fluorescence intensity changes in response to ISO treatment in BAs pre-treated with 2 μM TG, corresponding to time courses in Figure 6C. 38 μm × 38 μm (W × H). [file mmc10.jpg]

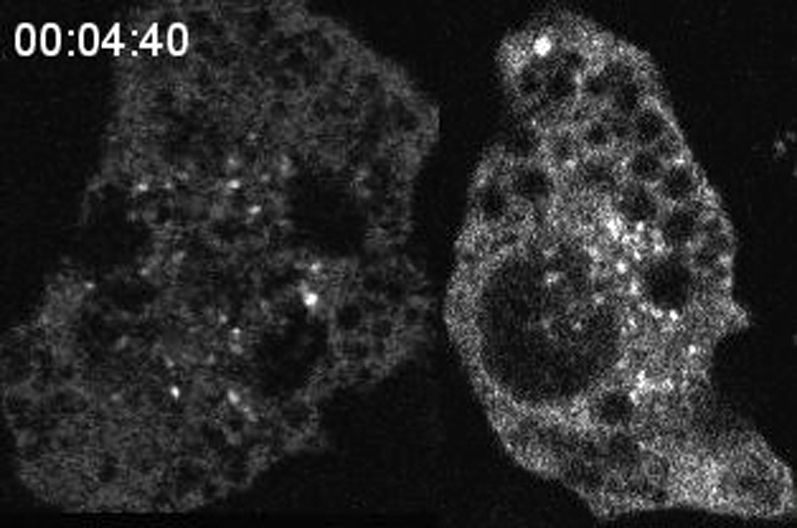

Supplement: Movie S10 — Mito-R-GECO (left) and ER-R-GECO (right) fluorescence intensity changes in response to ISO treatment and subsequent EGTA perfusion, corresponding to time courses in Figure S5C and E, respectively. 40 μm × 47 μm (left) and 31 μm × 46 μm (right) (W × H). [file mmc11.jpg]
