# Supplementary material for: Ca2+-associated triphasic pH changes in mitochondria during brown adipocyte activation
Source: Mol Metab. 2017 May 31;6(8):797–808. doi: 10.1016/j.molmet.2017.05.013 (PMC5518710; doi:10.1016/j.molmet.2017.05.013)
Supplement: Supplementary file 11 [file mmc1.pdf]

Supplementary Information

Supplementary Figures and Legends

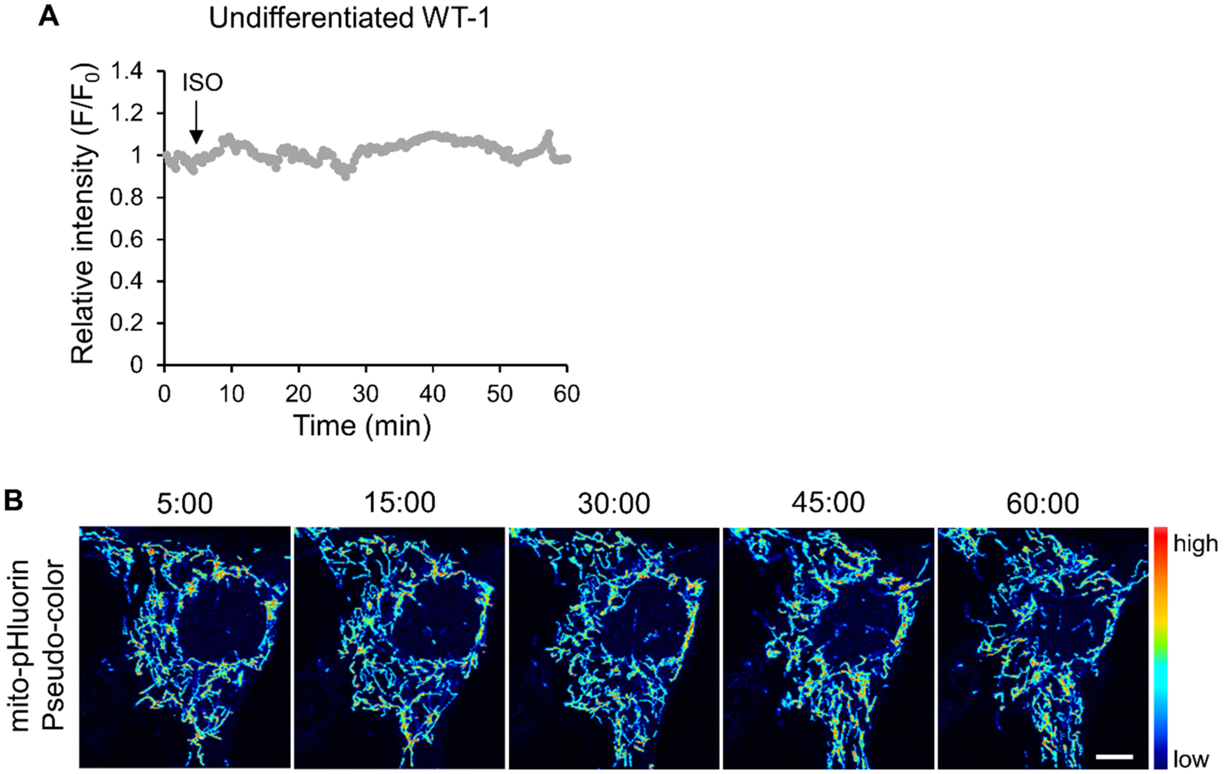

Figure S1: The effect of ISO on mitochondrial pH in undifferentiated WT-1 cells. (**A**) A representative time course showing the relative fluorescence intensity changes of mito-pHluorin in an undifferentiated WT-1 cell following ISO treatment over 60 min (n = 3). (**B**) Pseudo-color images of the cell quantified in A showing the fluorescence intensity changes of mito-pHluorin during ISO stimulation. See Movie S2. Scale bar, 10 μm.

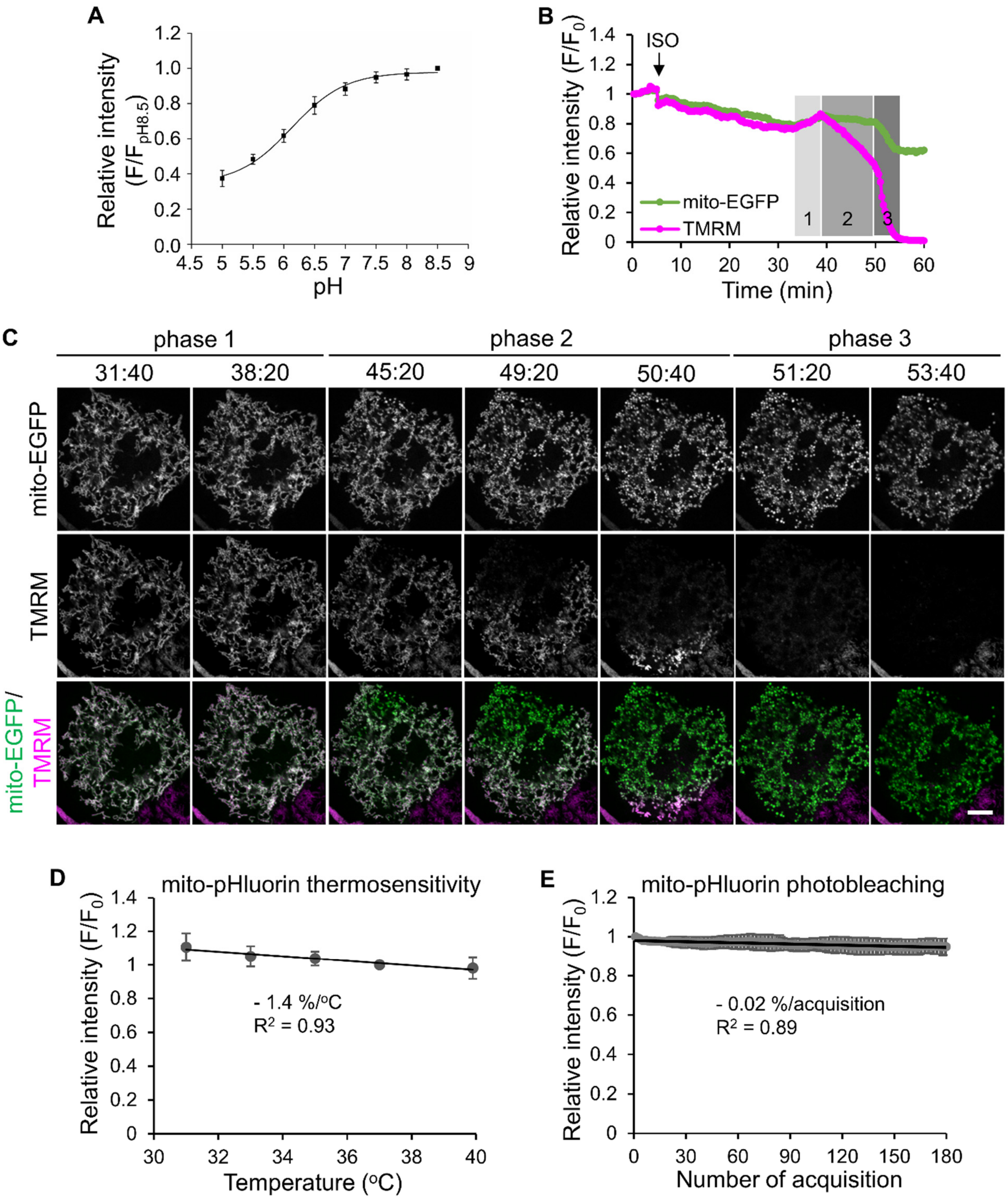

Figure S2: Examination of the influence of mitochondrial morphological changes and temperature sensitivity on intensities of fluorescent proteins. (**A**) Titration curve of mito-EGFP. The curve is the best fit of the relative fluorescence intensity versus pH, according to the

Henderson-Hasselbalch equation. (**B**) A representative time course showing the relative fluorescence intensity changes of mito-EGFP and TMRM in BAs following ISO stimulation (n = 3). Different phases are marked with shaded areas on the graph. (**C**) Images of the cell quantified in B. See Movie S3. Scale bar, 10 μm. (**D**) Fluorescence intensities of mito-pHluorin at different temperatures normalized to 37 °C. The temperature sensitivity of mito-pHluorin was determined by plotting the relative fluorescence intensity versus temperature. Data are mean ± SD (n = 8 cells). (**E**) BAs transfected with mito-pHluorin were subjected to imaging with the same illumination parameters as those stimulated with ISO, except that freerun rather than an interval of 20 sec was used. The fluorescence intensity of each acquisition was normalized to the first acquisition. Data are mean ± SD (n = 7 cells).

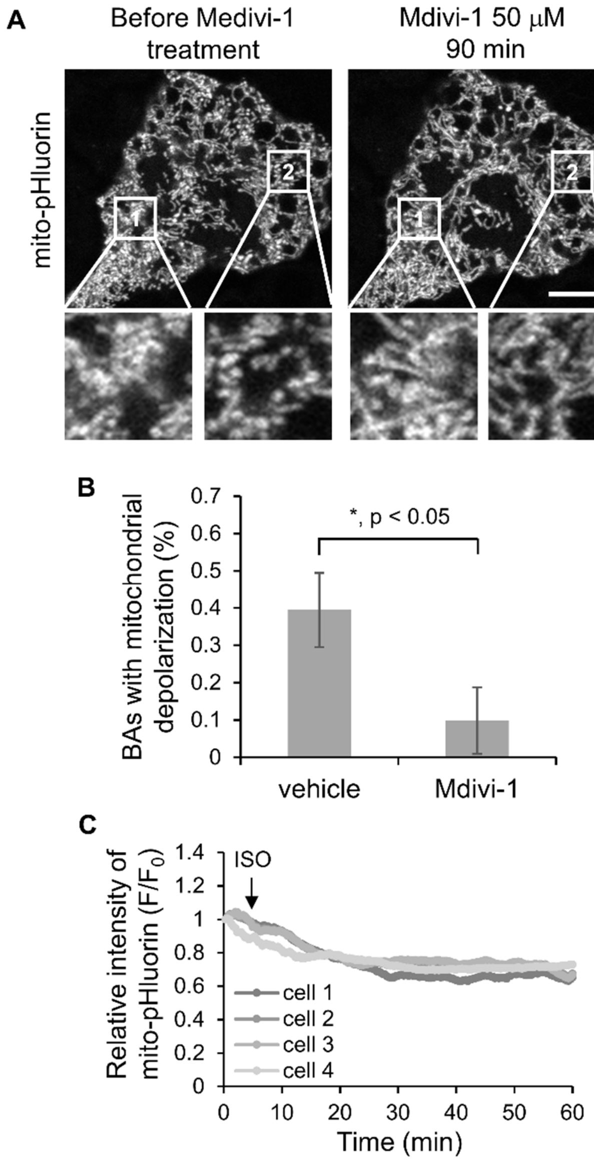

Figure S3: Examination of the pH changes in fission-resistant BAs. (**A**) BAs were treated with 50 μM Mdivi-1 for 90 min. Images showing the morphology of mitochondria before and after 90 min treatment of Mdivi-1. Scale bar, 10 μm. (**B**) BAs stained with TMRM were treated with vehicle or 50 μM Mdivi-1 for 60 min prior to ISO stimulation. Percentage of depolarized BAs in response to ISO stimulation were quantified. Data are mean ± SD (n = 3 independent dishes). (**C**) Representative graphs showing the relative fluorescence intensity changes of mito-pHluorin in ISO-stimulated BAs pretreated with Mdivi-1.

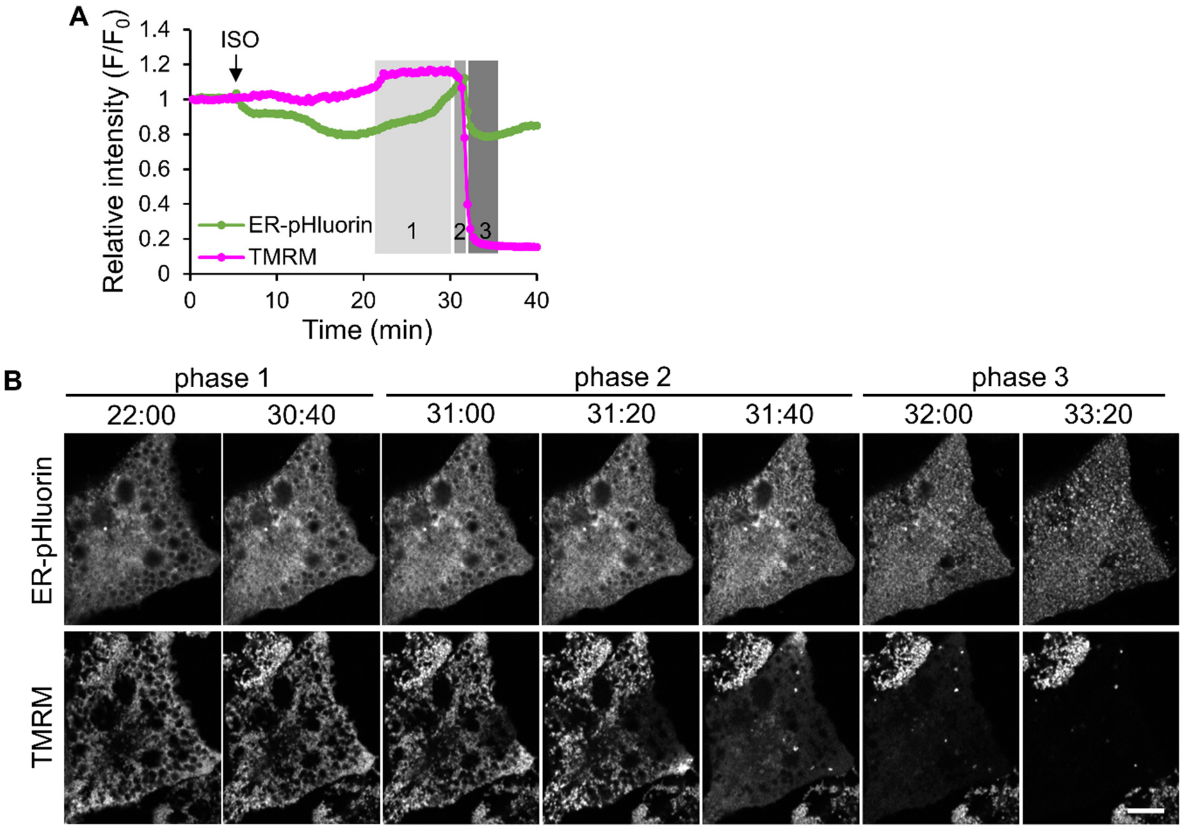

Figure S4: Characterization of the three phases in ER. (**A**) Representative time courses showing the changes in relative fluorescence intensity of ER-pHluorin and TMRM following ISO stimulation (n = 6). (**B**) Images of the cell quantified in A. By comparing to the intensity changes of TMRM, the three phases delineated in mitochondria can be applied in ER. Phase 1: pH increase in ER with a moderate increase in $\Delta\Psi_m$. Phase 2: wave of ER fragmentation and mitochondrial depolarization. Phase 3: pH decrease in ER with further mitochondrial depolarization. See Movie S6. Scale bar, 10 μm.

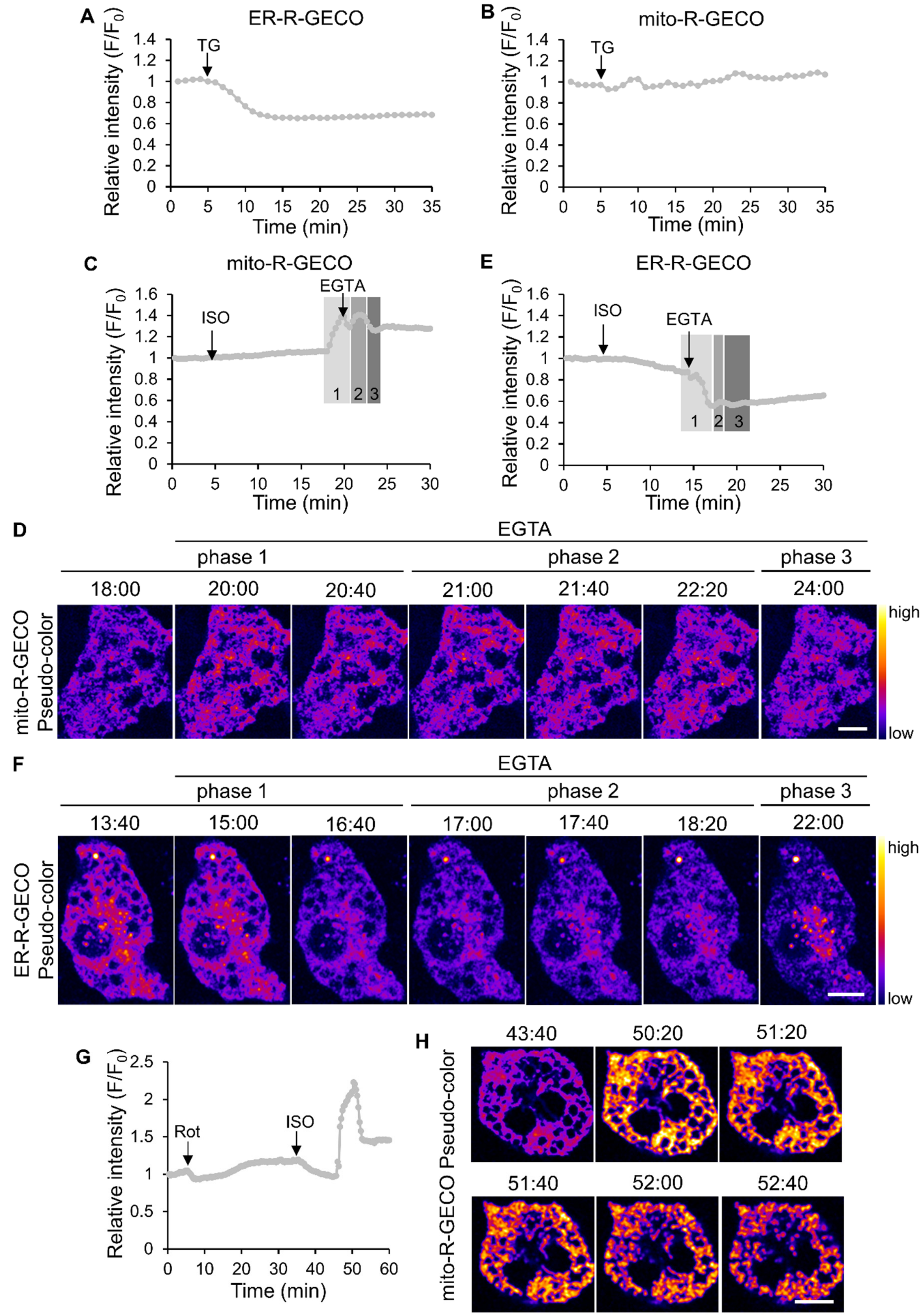
A
ER-R-GECO
TG
Relative intensity (F/F0)
Time (min)
B
mito-R-GECO
TG
Relative intensity (F/F0)
Time (min)
C
mito-R-GECO
ISO
EGTA
1 2 3
Relative intensity (F/F0)
Time (min)
E
ER-R-GECO
ISO
EGTA
1 2 3
Relative intensity (F/F0)
Time (min)
D
EGTA
phase 1
phase 2
phase 3
18:00
20:00
20:40
21:00
21:40
22:20
24:00
mito-R-GECO Pseudo-color
high
low
F
EGTA
phase 1
phase 2
phase 3
13:40
15:00
16:40
17:00
17:40
18:20
22:00
ER-R-GECO Pseudo-color
high
low
G
Rot
ISO
Relative intensity (F/F0)
Time (min)
H
43:40
50:20
51:20
51:40
52:00
52:40
mito-R-GECO Pseudo-color

Figure S5: Effect of chemical drugs on Ca^{2+} profiles in mitochondria and the ER. (**A, B**) Effect of thapsigargin (TG) on Ca^{2+} level changes. Representative time courses showing the relative fluorescence intensity changes of ER-R-GECO (**A**, n = 6) and mito-R-GECO (**B**, n = 5) following 2 μM TG treatment. (**C-F**) Effect of ethylene glycol tetraacetic acid (EGTA) on Ca^{2+} uptake in phase 2. Representative time courses showing the relative fluorescence intensity changes of mito-R-GECO (**C**, n = 3) and ER-R-GECO (**E**, n = 2) following ISO stimulation and subsequent 5 mM EGTA perfusion. Pseudo-color images (**D** and **F**) of the cell quantified in **C** and **E**, respectively. See Movies S10, left (for **D**) and right (for **F**). (**G** and **H**) Effect of rotenone (Rot) on Ca^{2+} wave generation. A representative time course showing the relative fluorescence intensity changes of mito-R-GECO following 30 min 5 μM Rot treatment and subsequent ISO stimulation (**G**, n = 3). Pseudo-color images (**H**) of the cell quantified in **G** showing mito-R-GECO intensity changes following ISO stimulation in Rot-pretreated BAs. See Movie S4, right. Scale bars, 10 μm.

Supplemental Experimental Procedures

Confocal microscopy

For confocal imaging of undifferentiated WT-1, cells were cultured and transfected with mito-pHluorin with FugeneHD (Promega, WI, USA). After overnight incubation with transfection mixture, cells were cultured for one more day before imaging. Images were captured at 20 sec intervals over 60 min.

For confocal imaging of ER-R-GECO/mito-R-GECO following TG treatment, BAs were treated with 2 μM TG at t = 5 min during time-lapse imaging. Images were captured at 1 min intervals over 35 min.

mito-EGFP plasmid construction and titration

To generate a mitochondrial matrix-localized EGFP, the mitochondria targeting signal sequence was inserted to the N-terminus of EGFP in pEGFP-C3 vector, generating pmito-EGFP-C3 (mito-EGFP). Titration of mito-EGFP was performed as described in the main text.

Determination of the temperature sensitivity of mito-pHluorin in living BAs

Temperatures of transfected BAs were maintained by a stage top incubator and measured by a digital thermometer (RX-450K, AS ONE, Japan). Images of BAs at different temperatures from high to low were captured sequentially.

Supplemental Movies and Legends

Movie S1. Mito-pHluorin fluorescence intensity changes in response to vehicle (left) or ISO (right) treatment in BAs, corresponding to time courses in Figure **1B**, left and right, respectively. 20 µm × 29 µm (left) and 36 µm × 72 µm (right) (W × H).

Movie S2. Mito-pHluorin fluorescence intensity changes in response to ISO treatment in undifferentiated WT-1 cells, corresponding to the time course in Figure **S1A**. 56 µm × 65 µm (W × H).

Movie S3. Mito-EGFP (left) and tetramethylrhodamine methyl ester (TMRM, right) fluorescence intensity changes in response to ISO treatment in BAs, corresponding to time courses in Figure **S2B**. 52 µm × 56 µm (W × H).

Movie S4. Mito-pHluorin (left) and mito-R-GECO (right) fluorescence intensity changes in response to 5 µM rotenone (Rot) treatment and subsequent ISO stimulation in BAs, corresponding to time courses in Figure **3A** and Figure **S5G**, respectively. 31 µm × 29 µm (W × H).

Movie S5. Mito-pHluorin (left) and mito-R-GECO (right) fluorescence intensity changes in response to ISO treatment in BAs, corresponding to time courses in Figure **4A**. 33 µm × 47 µm (W × H).

Movie S6. ER-pHluorin (left) and TMRM (right) fluorescence intensity changes in response to ISO treatment in BAs, corresponding to time courses in Figure **S4A**. 43 µm × 53 µm (W × H).

Movie S7. ER-pHluorin (left) and ER-R-GECO (right) fluorescence intensity changes in response to ISO treatment in BAs, corresponding to time courses in Figure **5B**. 33 µm × 46 µm (W × H).

Movie S8. ER-pHluorin (left) and ER-R-GECO (right) fluorescence intensity changes in response to ISO treatment in BAs pre-treated with 2 μM thapsigargin (TG), corresponding to time courses in Figure **6A**. 31 μm × 41 μm (W × H).

Movie S9. Mito-pHluorin (left) and mito-R-GECO (right) fluorescence intensity changes in response to ISO treatment in BAs pre-treated with 2 μM TG, corresponding to time courses in Figure **6C**. 38 μm × 38 μm (W × H).

Movie S10. Mito-R-GECO (left) and ER-R-GECO (right) fluorescence intensity changes in response to ISO treatment and subsequent EGTA perfusion, corresponding to time courses in Figure **S5C** and **S5E**, respectively. 40 μm × 47 μm (left) and 31 μm × 46 μm (right) (W × H).
